# Comparative mitogenomics indicates respiratory competence in parasitic *Viscum* despite loss of complex I and extreme sequence divergence, and reveals horizontal gene transfer and remarkable variation in genome size

**DOI:** 10.1186/s12870-017-0992-8

**Published:** 2017-02-21

**Authors:** Elizabeth Skippington, Todd J. Barkman, Danny W. Rice, Jeffrey D. Palmer

**Affiliations:** 1 Department of Biology, Indiana University, Bloomington, IN 47405 USA; 2 Department of Biological Sciences, Western Michigan University, Kalamazoo, MI 49008 USA; 3 Present address: Department of Bioinformatics and Computational Biology, Genentech Inc., 1 DNA Way, South San Francisco, CA 94080 USA

**Keywords:** Mitogenome, Mutation rate, Complex I, Mistletoes, Parasitic plants, Respiratory competence

## Abstract

**Background:**

Aerobically respiring eukaryotes usually contain four respiratory-chain complexes (complexes I-IV) and an ATP synthase (complex V). In several lineages of aerobic microbial eukaryotes, complex I has been lost, with an alternative, nuclear-encoded NADH dehydrogenase shown in certain cases to bypass complex I and oxidize NADH without proton translocation. The first loss of complex I in any multicellular eukaryote was recently reported in two studies; one sequenced the complete mitogenome of the hemiparasitic aerial mistletoe, *Viscum scurruloideum*, and the other sequenced the *V. album* mitogenome. The *V. scurruloideum* study reported no significant additional loss of mitochondrial genes or genetic function, but the *V. album* study postulated that mitochondrial genes encoding all ribosomal RNAs and proteins of all respiratory complexes are either absent or pseudogenes, thus raising questions as to whether the mitogenome and oxidative respiration are functional in this plant.

**Results:**

To determine whether these opposing conclusions about the two *Viscum* mitogenomes reflect a greater degree of reductive/degenerative evolution in *V. album* or instead result from interpretative and analytical differences, we reannotated and reanalyzed the *V. album* mitogenome and compared it with the *V. scurruloideum* mitogenome. We find that the two genomes share a complete complement of mitochondrial rRNA genes and a typical complement of genes encoding respiratory complexes II-V. Most *Viscum* mitochondrial protein genes exhibit very high levels of divergence yet are evolving under purifying, albeit relaxed selection. We discover two cases of horizontal gene transfer in *V. album* and show that the two *Viscum* mitogenomes differ by 8.6-fold in size (66 kb in *V. scurruloideum*; 565 kb in *V. album*).

**Conclusions:**

*Viscum* mitogenomes are extraordinary compared to other plant mitogenomes in terms of their wide size range, high rates of synonymous substitutions, degree of relaxed selection, and unprecedented loss of respiratory complex I. However, contrary to the initial conclusions regarding V. *album*, both *Viscum* mitogenomes possess conventional sets of rRNA and, excepting complex I, respiratory genes. Both plants should therefore be able to carry out aerobic respiration. Moreover, with respect to size, the *V. scurruloideum* mitogenome has experienced a greater level of reductive evolution.

**Electronic supplementary material:**

The online version of this article (doi:10.1186/s12870-017-0992-8) contains supplementary material, which is available to authorized users.

## Background

Almost all eukaryotes capable of carrying out aerobic respiration contain four multi-subunit electron-transfer complexes (complexes I-IV) that are more-or-less embedded in the inner mitochondrial membrane. Three of these complexes (I, III, and IV) translocate protons across this membrane to generate a proton gradient used by the mitochondrial ATP synthase (complex V) to phosphorylate ADP to ATP. In all respiring eukaryotes, the mitogenome contains genes for subunits of at least one of these complexes. Most often, as in all animals and virtually all plants, genes for subunits of complexes I, III, IV, and V are present in mitochondrial DNA. In several lineages of aerobic eukaryotes, complex I has been lost, with an alternative, nuclear-encoded NADH dehydrogenase known or thought to bypass complex I and oxidize NADH without proton translocation, while the apicomplexan relative *Chromera velia* was recently shown to have uniquely lost (for an aerobe) both complexes I and III [1].

In June, 2015, we reported the complete sequence of the mitogenome of the aerial hemiparasite (“mistletoe”) *Viscum scurruloideum* [2]. Most notable among several unusual features of this genome is its lack of the nine genes for complex I that are present in all other 300+ examined angiosperms [3, 4], whereas it contains a typical complement of plant mitochondrial genes for complexes II-V and for ribosomal RNA. Because functional transfer to the nucleus of all nine missing complex I (*nad*) genes is prohibitively unlikely [2], we concluded that *V. scurruloideum* represents the first reported case of loss of complex I in any multicellular eukaryote.

In December 2015, Petersen et al. [5] reported a partial complement of mitochondrial genes for *V. minimum* and *V. crassulae* and a complete mitogenome sequence for *V. album*, known as the common or European mistletoe. On two key issues, this study reached opposite conclusions than those published for *V. scurruloideum*. These conclusions were 1) that “mitochondrial genes encoding proteins of all [*Viscum*] respiratory complexes are lacking or pseudogenized” and 2) that the mitochondrial rRNA genes are either missing or so divergent in *Viscum* that their mitochondria “would be unable to perform protein biosynthesis unless all the missing rRNA[s]…are imported.”

These opposing conclusions, which were not discussed in the *V. album* paper [5], as it did not cite the *V. scurruloideum* study, could largely or entirely reflect a greater degree of reductive/degenerative evolution of the mitochondrial gene repertoire in *V. album* relative to *V. scurruloideum*. Alternatively, they could result primarily from interpretational and analytical differences between the two *Viscum* studies [2, 5]. Therefore, to address these issues, we have reannotated and reanalyzed the mitogenome of *V. album* using sequence-analysis parameter settings better suited for the high levels of sequence divergence in *Viscum* mitogenomes [2, 5–7] and compared the *V. album* genome to the *V. scurruloideum* genome. We show that the major conclusions reached in the *V. album* study [5] are incorrect: The two *Viscum* mitogenomes have a virtually identical complement of vertically inherited protein and rRNA genes, including a full set of rRNA genes and an essentially full set of complex II-V genes, with the *V. album* mitogenome containing 21 intact protein genes rather than the 12 reported by the *V. album* study [5]. We also show that the anomalously low level of divergence exhibited by two *V. album* genes is most likely the result of their recent acquisition via horizontal gene transfer (HGT), rather than evidence that they “may [be]…the only truly functional genes” in the *V. album* mitogenome. Finally, we report that the *V. album* and *V. scurruloideum* mitogenomes exhibit a remarkable 8.6-fold difference in size.

## Results and Discussion

### Reassessing mitochondrial protein gene loss in *Viscum album*

To identify protein and rRNA genes in the *V. album* mitogenome, Petersen et al. [5] compared it against local databases of mitochondrial protein and rRNA genes from 20 unnamed plant mitogenomes using BLASTX and BLASTN [8], respectively, with unspecified BLAST-parameter settings. Absence of BLAST high*-*scoring segment pairs (HSPs) was considered evidence for gene loss. This approach is problematic because extensive sequence divergence, as reported in both *V. album* and *V. scurruloideum* [2, 5], can obscure the underlying sequence similarity possessed by homologous genes [9]. The absence of HSPs can be indicative of gene loss, but at high levels of sequence divergence, and dependent on various parameter settings, BLAST searches frequently yield false negatives in homolog detection [10].

**Fig. 1:**
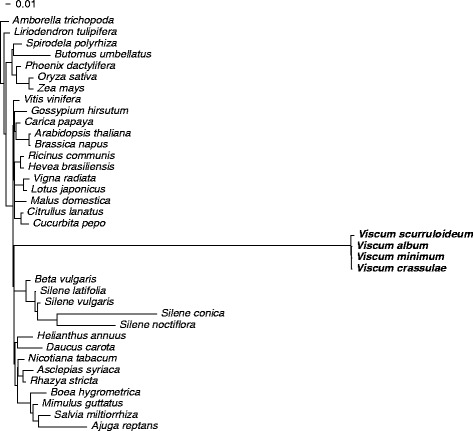
*Viscum* genes are extremely divergent relative to genes of other angiosperms with sequenced mitogenomes (see Fig. 3 for comparison to two divergent angiosperms, *Plantago* and *Pelargonium*, for which only a few gene sequences are available). A constrained topology was enforced for maximum likelihood (RAxML, GTRGAMMA) branch-length estimation on the basis of a concatenated alignment of all three codon positions of the nine best-conserved protein genes in *Viscum scurruloideum* (*atp1*, *atp6*, *atp9*, *ccmC*, *cob*, *cox1*, *cox2*, *cox3*, *rps12*; see [2])

We therefore reannotated the *V. album* mitogenome using two complementary strategies. First, the *V. album* genome was compared to mitochondrial gene sequences from 33 complete angiosperm mitogenomes (listed in Additional file 1: Figure S1) using BLASTN 2.2.28+ [8] with the following parameter settings: word size = 7, reward = 5, penalty = −4, gapopen = 8, and gapextend = 6. These sensitive settings allowed us to detect divergent gene sequences that went undetected in the published *V. album* annotation [5]. Second, the rRNA- and protein-gene sequences of the *V. scurruloideum* mitogenome [2] were compared to the *V. album* genome using BLASTN with default parameter settings. This provided additional power to delineate *V. album* mitochondrial gene and exon boundaries, as these genes are extremely divergent relative to genes of all examined non-*Viscum* angiosperms, but highly similar to those of *V. scurruloideum* (Fig. 1). Both strategies were needed because, although using the *V. scurruloideum* genes as BLAST queries is in general more effective, using this approach alone would have missed any genes present in *V. album*, but absent from *V. scurruloideum*.

**Fig. 2:**
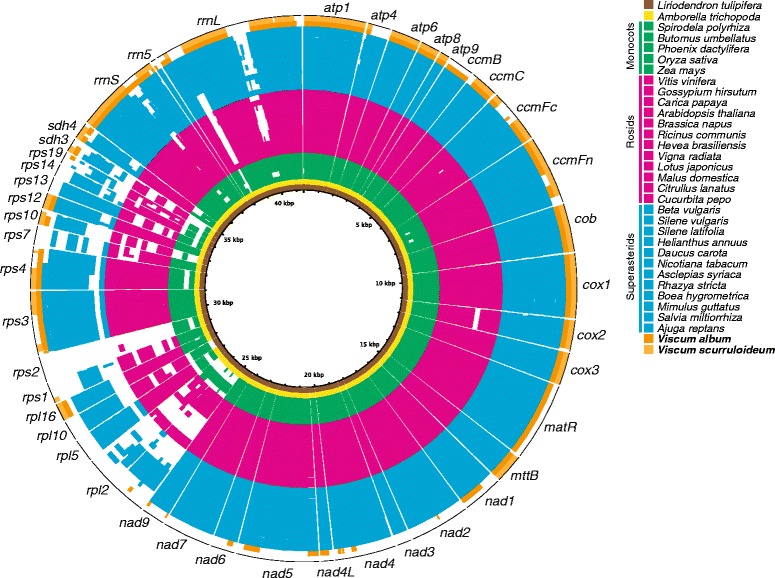
Evidence for virtually identical content of intact protein and rRNA genes in *V. album* and *V. scurruloideum*. Shown is a genome-wide BLAST comparison of angiosperm mitochondrial rRNA- and protein-coding sequences. Rings show high*-*scoring segment pairs (HSPs) yielded by BLASTn searches of the corresponding query mitochondrial genome against *Liriodendron* rRNA- and protein-coding sequences (total length = 41,475 bp) with the following parameter settings: evalue = 0.1, word_size = 7, reward = 5, penalty = −4, gapopen = 8 gapextend = 6, and perc_identity = 20. The outer ring (of thin black segments) displays the *Liriodendron* sequences shown to scale. Inner rings correspond to completely sequenced angiosperm mitochondrial genomes of magnolids (brown), monocots (*green*), rosids (*pink*), Santalales (*orange*), and superasterids excluding Santalales (*blue*). For the *Amborella* ring, only HSPs involving native *Amborella* genes are shown. This figure was produced using BRIG [43]

Angiosperm mitogenomes almost invariably share a set of 24 “core” protein genes, but can differ substantially in their inventories of a further 17 variably-present protein genes [3, 4, 11]. Contrary to Petersen et al. [5], but as expected based on the results of Skippington et al. [2] and other comparative studies among angiosperms [3, 11], BLAST searches yielded HSPs between the *V. album* mitogenome and most of the 24 core protein genes (Fig. 2). Using these HSPs as a starting point, we were able to annotate 15 of the 24 core protein genes as intact in *V. album*, 13 of which are likely to be functional (as described in the HGT section below, the *V. album ccmB* and *matR* genes were probably acquired recently via HGT and are unlikely to be functional). These include genes of complexes III (*cob*), IV (*cox1*, *cox2*, and *cox3*) and V (*atp1*, *atp4*, *atp6*, *atp8*, and *atp9*), as well as cytochrome c biogenesis proteins (*ccmB*, *ccmC*, *ccmFc*, and *ccmFn*), maturase *matR*, and protein transporter *mttB*. Notably, *atp4, atp8, ccmFc*, and *mttB* were missed in the original annotation [5], presumably due to their extensive sequence divergence (41.5%, 56.0%, 51.1%, and 63.2% nucleotide identity, respectively, with their counterparts in the *Liriodendron* mitogenome; Table 1). The only departure in the *V. album* mitogenome with respect to core genes is the apparent loss or pseudogenization of all nine *nad* genes (*nad1*, *nad2*, *nad3*, *nad4*, *nad4L*, *nad5*, *nad6*, *nad7*, and *nad9*). Although this is not at all unexpected in light of the loss of respiratory complex I in *V. scurruloideum* (for comprehensive discussion of this loss, see Skippington et al. [2]), it nonetheless provides useful confirmation. In particular, the fact that the mitogenome of *V. album* contains clearly pseudogenized remnants of most *nad* genes, whereas that of *V. scurruloideum* contains no such traces, further rules out the already remote possibility that the latter species somehow contains functional but essentially undetectable forms of these genes. Because our annotation of the *V. album* mitogenome conflicts with the original conclusion [5] that “mitochondrial genes encoding proteins of all respiratory complexes are lacking or pseudogenized,” we will discuss evidence for the functionality of complexes II-V in a separate section.

**Table 1:** Protein-gene divergence in *Viscum album* (Va) vs. *Liriodendron tulipifera* (Lir) and Va vs. *V. scurruloideum* (Vs)

Gene	Coding sequence length (nt)			% nucleotide identity^a^		Va- Vs syn. and nonsyn.^b^		
	Lir	Va	Vs	Va vs. Lir	Va vs. Vs	d_N_	d_S_	d_N_/d_S_
*atp9*	225	225	219	82.7	91.8	0.044	0.216	0.20
*rps12*	375	417	411	68.0	98.3	0.010	0.048	0.21
*atp1*	1527	1536	1536	73.1	99.8	0.001	0.004	0.22
*cox1*	1572	1602	1602	81.4	99.6	0.003	0.010	0.24
*cox3*	795	738	795	75.1	99.2	0.006	0.017	0.33
*rps3*	1554	1455	1518	50.4	97.6	0.018	0.044	0.41
*ccmFc*	1326	1305	1218	51.1	96.3	0.029	0.067	0.43
*sdh3*	282	222	186	54.8	97.3	0.022	0.047	0.47
*atp6*	720	915	915	75.5	99.0	0.008	0.016	0.51
*ccmFn*	1683	1776	1467	51.8	96.7	0.029	0.051	0.56
*mttB*	720	765	789	63.2	96.9	0.029	0.042	0.68
*atp4*	558	471	474	41.5	98.5	0.014	0.020	0.69
*cob*	1179	1209	1221	74.4	99.3	0.006	0.008	0.73
*rps4*	1035	1005	1224	51.8	96.1	0.037	0.050	0.75
*rpl16*	432	378	378	63.2	98.4	0.015	0.019	0.79
*ccmC*	699	654	654	65.9	99.5	0.004	0.005	0.84
*atp8*	474	492	483	56.0	98.3	0.016	0.019	0.85
*cox2*	762	750	750	76.3	99.6	0.005	0.000	n.a.
*rps10*	357	273	273	59.4	99.6	0.006	0.000	n.a.
All genes^c^	16275	16188	16113	64.4	98.2	0.015	0.029	0.50

^a^Identity calculations exclude gap positions

^b^Synonymous (d_S_) and nonsynonymous (d_N_) site divergence

^c^Values are from a concatenate of all 19 protein genes

Petersen et al. [5] identified only one (*rps12*) of the 17 genes of highly variable presence in angiosperms. Our analysis also found *rps12*, as well as five genes (*rpl16*, *rps3*, *rps4*, *rps10*, and *sdh3*) that were classified as missing [5]. In *V. scurruloideum*, we originally annotated *sdh3* as a pseudogene due to its truncated length (186 nt compared to 282 nt in *Liriodendron*) [2], but in light of high sequence identity (97.3%) with its 222 nt counterpart in *V. album*, we have reannotated *sdh3* as putatively functional in both *Viscum* species. Altogether, we identified 21 putatively functional protein genes in the *V. album* mitogenome assembly (Additional file 1: Table S1 and Figure S1), compared to the 12 originally reported [5].

### A complete set of functional rRNAs in *V. album*

Petersen et al. [5] state that “we were unable to locate 5S rRNA in any of the three *Viscum* species, and the recovered 26S rRNA-like sequences are so divergent that the genes are unlikely to be functional” and that “The identified sequences of the 18S rRNA genes are also very different from those of all other seed plants suggesting that they too are not functional.” However, there was no evidence provided to support these claims regarding the 18S and 26 rRNAs, including any quantitative sense – in terms of either phylogenetic or comparative sequence analysis – of the level of divergence exhibited by the *V. album* rRNA sequences. We readily identified intact and almost certainly functional genes in *V. album* for the large subunit (LSU), small subunit (SSU) and 5S rRNAs (*rrn*L, *rrn*S, *rrn*5, respectively) (Fig. 2). It is true that all three of these genes are divergent in the context of angiosperm evolution (Additional file 1: Figure S2), with *rrn*L, *rrn*S, and *rrn*5 sharing only 63%, 70%, and 53% identity, respectively, with their counterparts in the relatively ancestral-like and slowly evolving *Liriodendron* mitogenome. However, there is a remarkable level of variation in the size, sequence, and structure of mitochondrial rRNAs across eukaryotes, a level that far exceeds the variation seen in rRNAs across prokaryotes, nuclear genomes, and plastid genomes, and a level against which the *Viscum* divergence pales. For example, the SSU and LSU rRNAs of animal mitochondria vary in size by a factor of 4.0 (513–2,036 nt) and 6.6 (529–3,487 nt), respectively [12]. A broad diversity of mitochondrial genomes do not even have complete SSU and LSU rRNA genes, with the gene fragments sometimes extensively scrambled in the genome, and with as many as 27 fragments for both genes combined in *Plasmodium falciparum* (e.g., [13, 14]). Despite this divergence, those regions of the mitochondrial SSU and LSU rRNAs that assemble to form the catalytic core of each ribosomal subunit are conserved in all known mitochondria [14, 15].

Furthermore, we superimposed the *V. album* SSU and LSU rRNA sequences onto the secondary structures of mitochondrial SSU and LSU rRNAs from the angiosperms *Oenothera berteriana* and *Zea mays*, respectively (Additional file 1: SI Appendix, Figures S3 and S4, respectively). This shows that the *V. album* rRNAs share extensive sequence identity with the highly conserved regions of large and small subunit rRNAs and strongly implies the existence of rigid functional and structural constraints. We previously carried out similar analyses for *V. scurruloideum* that supported the functionality of its rRNAs [2]. Very few of the mutations in the *V. scurruloideum* rRNAs correspond to base-pairing nucleotides of the secondary structures, and most have been accompanied by compensatory base-pair changes (see Additional file 1: Figure S4 and S5 of [2]). The 18S rRNA of *V. scurruloideum* has 10 compensatory base-pair changes relative to reference *Oenothera berteriana*, while the 23S rRNA has 23 compensatory changes relative to *Zea mays*. The high level of sequence conservation between the *V. album* and *V. scurruloideum* SSU and LSU rRNAs (95% and 91% identity, respectively, gaps excluded) is also consistent with their being functional.

A full 5S rRNA alignment is shown in Additional file 1: Figure S5. The small size and/or complex secondary structures of mitochondrial 5S rRNA make identification notoriously difficult [16]. Indeed, we originally annotated only a 38 nt-long sequence as 5S rRNA in *V. scurruloidum* [2] because clear gene boundaries were difficult to ascertain. Pairwise alignment of this short sequence to the *Liriodendron* 5S rRNA yielded 87% identity, excluding gaps [2]. Here, using secondary structure analysis (Additional file 1: Figure S6) and sequence conservation (100% identity, in fact) between the two *Viscum* sequences, we were able to extend the annotated gene length to 118 nt in both species (as compared to 112–126 nt in most angiosperms). A recent study that used a mitochondrion-specific covariance model to screen mitochondrial genome sequences for 5S rRNA identified more than 50 previously unrecognized homologs in mitochondrial genomes of various stramenopiles, red algae, cryptomonads, malawimonads and apusozoans and also showed that 5S rRNAs have a much larger structural variability than previously thought [16]. However, the divergence of the 5S rRNAs discovered by Valach et al. [16] far exceeds the divergence of the *Viscum* 5S rRNAs relative to other angiosperms.

Six sequences were identified in the *V. album* mitogenome that could be folded into a typical tRNA structure (Additional file 1: Figure S7). These sequences correspond to two different copies each of *trnW (cca)* and *trnM (cau)*, and one copy each of *trnK (uuu)*, and *trnG (ggc)*. Although the *V. album* mitogenome contains far fewer types of tRNA genes than most other angiosperms, even greater reduction in tRNA gene content (two, three, and three, respectively) has been reported for the *S. conica*, *S. noctiflora,* and *V. scurruloideum* mitogenomes [2, 17]. Moreover, several lineages of non-plant eukaryotes, including *Trypanosoma brucei*, *Leishmania tarentolae*, and *Plasmodium falciparum*, have no tRNA genes left in their mitogenome and are thus entirely dependent on import of nuclear-encoded tRNAs [18–20].

Petersen et al. [5] state that “*Viscum* mitochondria would be unable to perform protein biosynthesis unless all the missing rRNA, ribosomal proteins and tRNAs are imported”. However, we are unaware of any precedent for the mitochondrial import of SSU and LSU rRNAs, nor, as described above, is there are reason to posit such import in *Viscum*. In contrast, many diverse eukaryotes import most or all of their mitoribosomal proteins and/or tRNAs [21]. Angiosperm mitoribosomes contain at least 63 proteins, 48 of which were encoded by the nucleus of the ancestral angiosperm, with the genes for another 15 proteins having been functionally relocated to the nucleus numerous times during angiosperm evolution [3]. Indeed many lineages of angiosperms have evolved smaller sets of mitoribosomal protein genes than the five present in *V. album* and *V. scurruloideum* (Additional file 1: Figure S1; [3, 17]). In conclusion, we see no reason to think that *V. album* mitochondria are either incapable of carrying out protein synthesis or require the unprecedented import of translational components.

### Reevaluating respiratory competence in the face of exceptional sequence divergence: evidence for a full set of functional complex II-V genes in *V. album*

Phylogenetic analysis (Additional file 1: Figure S2) of the 21 protein genes present in V. *album* shows that, except for *matR* and *ccmB* (see next section), all of these genes exhibit exceptionally high levels of divergence relative to most other angiosperms, including the two other sampled Santalales, *Comandra umbellata* (Comandraceae) and *Amyema beccarri* (Loranthaceae). Almost all of this divergence is located on the branch leading to the last common ancestor of *Viscum* (Table 1, Fig. 1, and Additional file 1: Figure S2). Our previous analyses of *V. scurruloideum* indicated that this divergence is largely a consequence of a highly elevated rate of synonymous substitutions [2]. Given the high levels of divergence observed in *V. scurruloideum*, we raised – and answered – the question of whether the most divergent *V. scurruloideum* genes are potentially functional [2]. Because the highly divergent protein genes shared by *V. album* and *V. scurruloideum* are extremely similar (12 of 19 are >98% identical in nucleotide sequence, and all but one are >96% identical; see Table 1), and because Petersen et al. [5] inferred that all five respiratory and ATP-synthesis complexes are defunct in *V. album*, it is prudent to revisit and extend our arguments for the functionality of *Viscum* protein genes.

As in *V. scurruloideum*, the 19 divergent protein genes in *V. album* are, for the most part, encoded by ORFs of moderate to considerable length (Table 1). Thirteen are of equal or greater length than their counterparts in *V. scurruloideum* (Table 1). Considering the highly accelerated evolution of *Viscum* mitochondrial sequences, we previously argued that premature stop codons should have been introduced into most if not all *V. scurruloideum* genes that were not functional [2]. Indeed we showed that the probabilities of such ORFs being maintained by chance alone are extremely low [2]. By extension, therefore it is likely that the *V. album* ORFs are, by and large, functional, because they are all comparable in length to those found in *V. scurruloideum* (Table 1).

To further assess the level of divergence and functionality of *Viscum* protein genes, we estimated *d*_*S*_ and *d*_*N*_ values (Table 1, Additional file 1: Figure S8). Wherever possible, we included *V. scurruloideum*, *V. minimum*, and *V. crassulae* homologs in *d*_*S*_ and *d*_*N*_ trees. The patterns of divergence in the *d*_S_ trees (Additional file 1: Figure S8) are similar to those in the trees based on all three codon positions (Additional file 1: Figure S2). For almost all genes, the longest *d*_S_ branch is the one leading to the last common ancestor of *Viscum*, and the longest root-to-tip branches are those leading to the sampled *Viscum* species (Additional file 1: Figure S8). As previously concluded [2], this divergence is consistent with a highly elevated rate of synonymous substitutions operating across the mitogenome in *Viscum*. There is comparatively little divergence among the four sampled *Viscum* species, indicating that most nucleotide substitutions occurred in the *Viscum* lineage before these species diverged (Additional file 1: Figure S8).

The *d*_*N*_ values on the branch leading to *Viscum* are also highly elevated relative to other angiosperms (Additional file 1: Figure S8). Moreover, in a number of gene trees (most notably, those for *atp1*, *atp6*, *ccmC*, *cob*, *cox1*, *cox2*, and *cox3*) *d*_*N*_/*d*_*S*_ values are markedly higher on the ancestral *Viscum* branch compared to other angiosperm branches with high *d*_*S*_ values (e.g., those leading to *Silene noctiflora* and *S. conica*). Furthermore, excluding *cox2* and *rps10*, which have no synonymous-site differences between *V. album* and *V. scurruloideum*, the average pairwise *d*_*N*_/*d*_*S*_ value for *V. album/V. scurruloideum* is 0.52 (Table 1), which is also unusually high. Importantly, however, in all cases *d*_*N*_/*d*_*S*_ for *V. album/V. scurruloideum* is below 1.0, ranging from 0.20 to 0.85 (Table 1), which again suggests that most if not of all these protein genes are functional. These findings, together with those previously reported [2], suggest that many protein genes in *Viscum* are evolving under relaxed selection relative to most plants. To formally test for changes in selective strength, we used RELAX [22], a codon-based, branch-site random-effects method, to test the nine best conserved [2] *Viscum* protein genes (excluding *matR* and *ccmB*, see next section) for changes in selective strength. We find that selection is significantly relaxed along the *Viscum* branches in eight of the nine gene sets (selection intensity *k* = 0.17–0.70; Table S2).

**Fig. 3:**
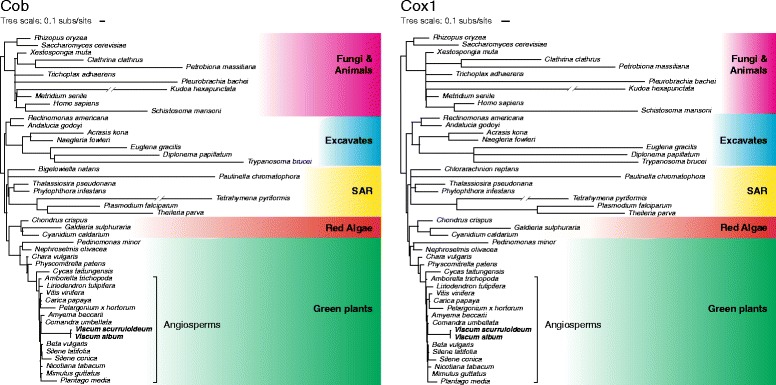
The high divergence of Cob and Cox1 in *Viscum* relative to other angiosperms pales in comparison to the extreme divergence of these proteins in many diverse lineages of eukaryotes. A constrained topology was enforced for protein maximum likelihood (RAxML, mtRev + G + I) branch-length estimation. Note that angiosperm sampling was deliberately chosen to include representatives of the three most divergent lineages of angiosperm mitogenomes besides *Viscum.* The three representatives are *Pelargonium x hortorum* [44], *Silene conica* [17], and *Plantago* rugelii [45]. See also Fig. 1 and Additional file 1: Figure S2 for angiosperm-only phylogenetic trees that include both *Viscum* and *S. conica* (and the high-rate *S. noctiflora,* too). *Tetrahymena pyriformis* and *Kudoa hexapunctata* branch lengths are shown reduced by a factor of 2

Finally, because it was implied [5] that the level of sequence divergence exhibited by *V. album* respiratory and ATP-synthesis genes is incompatible with them being functional, we sought to provide a broader phylogenetic context for this divergence. We therefore constructed protein trees for complex III protein Cob (cytochrome b) and complex IV protein Cox1 (cytochrome oxidase subunit 1), two of the best conserved proteins involved in respiration and ATP synthesis, using sequences chosen to reflect the breadth of Cob and Cox1 sequence diversity in eukaryotes. Although these two proteins are unusually divergent in *Viscum* relative to other flowering plants (Figs. 2 and 3), this divergence pales in comparison to their divergence in many other eukaryotes, including several groups within animals alone (Fig. 3). For example, Cob and Cox1 from the myxozoan *Kudoa hexapunctata*, which harbors the most divergent animal mitogenome characterized to date [23], are only 28% and 36% identical, respectively, to those of the slowly evolving sponge *Xestospongia muta*. In contrast, the *Viscum album* Cob and Cox1 proteins are 69% and 84% identical to homologs from the slowly evolving angiosperm *Liriodendron tulipifera*. This broader perspective supports the hypothesis that, apart from the undisputed loss of all complex I genes, the mitogenomes of *V. album* and other *Viscum* species possess the same basic set of *functional* respiratory and ATP-synthesis genes found in all other angiosperms examined to date.

### Horizontal gene transfer of *ccmB* and *matR*

**Fig. 4:**
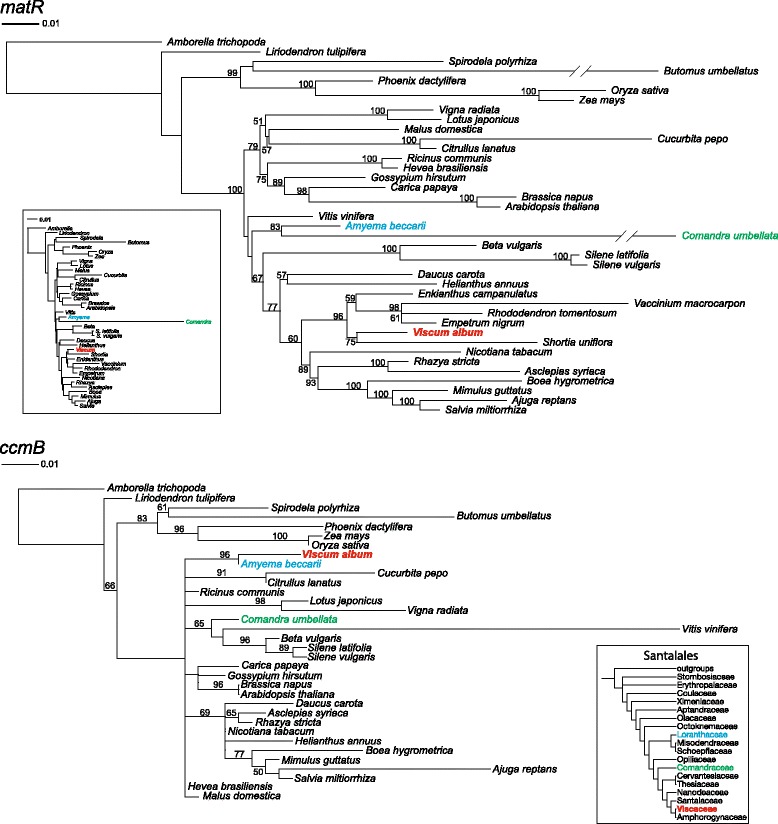
Evidence for horizontal origin of the *matR* and *ccmB* genes in *V. album* based on maximum-likelihood analysis of all-position nucleotide alignments. Note that *V. scurruloideum* lacks both of these genes. Bootstrap values >50% are shown. The Santalales phylogeny shown inset in the *ccmB* tree is the current best estimate of relationships among these plants [6, 7]

In contrast to the extremely divergent gene sequences typically found in *Viscum* (Fig. 1 and Additional file 1: Figure S2), the *V. album ccmB* and *matR* sequences are, as noted by Petersen et al. [5], unexceptional in their level of sequence divergence (Fig. 4). This observation, together with the absence of *ccmB* and *matR* in *V. scurruloideum* [2] and their apparent absence in *V. minimum* and *V. crassulae* [5], suggests that these genes were reacquired recently, on the branch leading to *V. album*. To verify that these two gene sequences belong to *V. album* rather than being the result of contamination, we used PCR to amplify them from an independent sample of *V. album* DNA. Sequencing of these PCR products recovered identical *ccmB* and *matR* sequences to those reported in the *V. album* paper [5], thereby verifying the original report.

Consistent with the above observations, phylogenetic analyses indicate that *V. album* acquired *matR* and *ccmB* via HGT, probably from two different donors (Fig. 4). The *matR* tree placed *V. album* within Ericales with strong support (96% bootstrap). This order contains, among others, blueberries (*Vaccinium*), primroses (*Primula*), and tea (*Camellia*) (Fig. 4). To assess confidence in the aberrant *matR* topology shown in Fig. 4, we used the approximately unbiased (AU) test [24] to compare it to an alternative topology, of strictly vertical *matR* transmission, in which *V. album matR* was constrained to be sister to *Comandra matR* (see inset cladogram in Fig. 4). This alternative tree was rejected with a *P* value of 1e-06. These phylogenetic results, in conjunction with the divergence and distributional data, strongly indicate that *V. album* acquired *matR* recently by HGT, most likely from a member of the Ericales. *Viscum album,* which is naturally widespread in Europe and central and western Asia*,* is known to parasitize no fewer than 452 species from 44 families [25]. Consistent with the phylogenetic results, *V. album* has been found to parasitize introduced members of two Ericales families (Ericaceae and Ebenaceae). Although these Ericales species do not occur within the current native range of *V. album* [25], ancestral range overlaps of a parasite and its hosts are difficult to infer in general, and all the more so given that *V. album* currently parasitizes so many diverse hosts.

The *ccmB* tree placed *V. album* as sister to another member of Santalales, *Amyema*, also with strong support (96% bootstrap). This placement is not congruent with Santalales phylogeny [6]; in the context of our sampling, *Viscum* should instead appear as sister to *Comandra* (Fig. 4). An alternative topology of strictly vertical transmission of *ccmB*, i.e., in which *V. album ccmB* was constrained as sister to *Comandra ccmB*, was rejected with a *P* value of 4e-4. These phylogenetic results, taken together with the divergence and distributional findings, strongly indicate that *V. album* acquired its *ccmB* gene by recent HGT from another lineage within Santalales. *Viscum album* is well known [25] to be hyperparasitic on another European aerial hemiparasite, *Loranthus europea,* a member of the same family (Loranthaceae) as *Amyema*. Thus, *V. album* may have acquired *ccmB* from a host plant, itself a parasite.

The *V. album* study [5] noted that the anomalously low level of sequence divergence exhibited by *ccmB* and *matR* “may indicate that they are the only truly functional genes” in the mitogenome. However, the phylogenetic and distributional evidence presented above suggest otherwise: The presence and anomalously low divergence of these two genes in *V. album* are most likely the result of two recent HGT events. Accordingly, the intactness of these genes in *V. album*, rather than being evidence for functionality, is probably simply a reflection of their limited residency in the *V. album* mitogenome, i.e., they have not been present in *V. album* long enough to incur pseudogenizing mutations.

### Enormous difference in mitogenome size between *V. album* and *V. scurruloideum*

At 565 kb, the *V. album* mitogenome [5] is far larger (by a factor of 8.6) than that of its closest sequenced relative, *V. scurruloideum* [2]. Much of this difference is presumably due to genome compaction in *V. scurruloideum*, which at 66 kb is the smallest (by over 3-fold) angiosperm mitogenome sequenced to date [2], whereas 565 kb is close to the median size of currently examined angiosperm mitogenomes (Additional file 1: Figure S1). This level of within-genus variation in mitogenome size is unusual for any group of eukaryotes, even angiosperms, long known to sustain massive and enigmatic changes in mitogenome size [17, 26]. Nine of the 10 other multiply sampled angiosperm genera exhibit less than two-fold variation in mitogenome size (http://www.ncbi.nlm.nih.gov/genome/organelle/), while the *Viscum* size difference exceeds that of all but one of the seven other angiosperm *families* for which multiple genera have been sampled. Even more exceptional than *Viscum* is the genus *Silene* (Caryophyllaceae), in which mitogenome size varies by a factor of 45 (from 253 kb to 11.3 Mb) among four examined species [17].

## Conclusions

*Viscum* mitogenomes are extraordinary compared to those of other angiosperms. Our reannotation and reanalysis of the *V. album* mitogenome has confirmed that the unprecedented loss of respiratory complex I in a multicellular organism is not restricted to *V. scurruloideum* [2] and probably occurred sometime prior to the divergence of this species and *V. album* (and also the other two *Viscum* species examined [5]). As well, both *Viscum* genomes are exceedingly divergent at the sequence level due to a combination of highly elevated synonymous substitution rates and relaxed selection. *Viscum scurruloideum* possesses other unusual properties that are either not shared by *V. album* (i.e., a highly miniaturized genome) or remain uninvestigated (i.e., unusually high levels of recombination across short repeats and of sublimons) [2].

Although the *V. album* mitogenome is extraordinary in certain ways, we find no evidence that it has sustained unprecedented events of gene loss or disabling divergence involving mitochondrial rRNAs or proteins fundamental to respiratory complexes III and IV and the mitochondrial ATP synthase. Instead, the presence in both *Viscum* mitogenomes of the same complement of essentially full-length respiratory genes that are all under some level of purifying selection, coupled with full-length rRNA sequences that exhibit compensatory mutations and canonical predicted secondary structures, indicates that respiratory and translational functions are almost certainly maintained in the *Viscum* lineage. Our findings emphasize the critical importance of conducting BLAST homology searches in highly divergent sequences with appropriately sensitive parameter settings in order to achieve accurate annotations.

The results of this study point to several directions for future research. First, the occurrence of multicellular aerobic respiration without complex I points to a need for additional functional analysis of the role of NADH oxidation. Second, the *Viscum* lineage exhibits a fascinating combination of sequence divergence and repeat-mediated recombination potential that could allow future studies to uncover mechanisms of genome size variation. Finally, the discovery of HGT in the mitogenome of *V. album* – one of the most familiar and common parasitic plants – may enable it to serve as a tractable system in which to study the dynamics of foreign sequence acquisition and integration.

## Methods

### Gene annotation

The two strategies used to detect protein and rRNA genes in the *V. album* mitogenome are described in the first section of Results and Discussion. tRNA genes were predicted using tRNAscan version 1.23 [27].

### Phylogenetic analysis and evolutionary rate estimation

Throughout this paper, *Viscum* gene divergence is reported with respect to *Liriodendron.* This is because the extraordinarily low genome-wide silent substitution rate [28] of the *Liriodendron* mitogenome makes it an ideal reference genome for comparative sequence analyses in angiosperms. All alignments were made using MAFFT v. 7.130b [29] with the L-INS-I option. All protein-gene sets were initially aligned at the amino acid level and then either analyzed as such (for the eukaryote-wide analyses shown in Fig. 2) or computationally converted to nucleotide alignments using PAL2NAL [30] such that the resulting arrangement of nucleotide triplets reflected the corresponding protein alignment. Codons empirically determined to undergo mitochondrial RNA editing in at least one of eight angiosperms (*Arabidopsis thaliana* [31]*, Beta vulgaris* [32]*, Brassica napus* [33]*, Citrullus lanatus* [34]*, Cucurbita pepo* [34]*, Oryza sativa* [35], *Silene latifolia* [36], and *Vitis vinifera* [37]) or predicted (using PREP-Mitochondrial [38] with a stringency setting of 0.2) to undergo RNA editing in *V. scurruloideum, Amyema,* or *Comandra* were excluded from all nucleotide analyses. All phylogenetic trees constructed from nucleotide alignments were estimated with RAxML v. 7.2.8 [39] using the generalized time-reversible (GTR) model with gamma correction for among-site rate variation and 10 starting trees. Support for nodes was assessed using 1,000 bootstrap replicates. The topologically constrained amino-acid trees shown in Fig. 3 were also estimated with RAxML, but under the mtRev + G + I model.

PAML’s codeml [40] was used to estimate *d*_*S*_ and *d*_*N*_ values as described previously [2]. A simplified Goldman–Yang codon model was used with separate branch *d*_*N*_/*d*_*S*_ ratios (ω) that allowed for the following seven sets of branches: the *V. scurruloideum* branch, the *V. album* branch; the *V. minimum* branch, the *V. crassulae* branch; the ancestral *Viscum* branch; the *S. conica* and *S. noctiflora* branches (one set); and all remaining branches.

To test for differences in selection pressure between *Viscum* and non-*Viscum* branches of protein-gene trees, we used a codon-based model-testing framework implemented in RELAX [22], available on Datamonkey [41].

### Alternative topology tests

The CONSEL package [42] was used to calculate the approximately unbiased (AU) *P* values for unconstrained and constrained trees. To generate constrained ML trees for *matR* and *ccmB*, we required *V. album* to be sister to *Comandra*, and conducted ML searches under this constraint using RAxML v. 7.2.8 [42].

### PCR and DNA sequencing

Total DNA from *V. album* was extracted using the DNeasy kit (Qiagen) from 20 mg of leaf tissue collected from a plant growing on an apple tree in Oviedo, Spain. We performed PCR to amplify *matR* and *ccmB* from *V. album* using the following primers: *matR*-forward, GTTTTCACACCATCGACCGACATCG; *matR*-reverse, CGCGGCACCTGTAGTAGGACAGAGGA; *ccmB*-forward, CATGTCATTCCCATTTAGGTCCG; and *ccmB*-reverse: GGTGAAGTGGTTGGATTTAGCG. Thermal cycling conditions included an initial hold at 94 °C for 2' 30" followed by 35 cycles of 94 °C for 30", 50 °C for 1', and 72 °C for 1' 30". Amplification products were purified using the QIAquick PCR Purification kit (Qiagen), and both strands were sequenced.

## Additional file

Additional file 1: Supplementary material for “Comparative mitogenomics indicates respiratory competence in parasitic *Viscum* despite loss of complex I and extreme sequence divergence, and reveals horizontal gene transfer and remarkable variation in genome size”. Tables S1 to S2 and Figures S1 to S8. (PDF 12394 kb)

## Abbreviations

AU: Approximately unbiased
GTR: Generalized time-reversible
HGT: Horizontal gene transfer
HSPs: High-scoring segment pairs
LSU: Large subunit
SSU: Small subunit
