# Supplementary material for: Comparative mitogenomics indicates respiratory competence in parasitic Viscum despite loss of complex I and extreme sequence divergence, and reveals horizontal gene transfer and remarkable variation in genome size
Source: BMC Plant Biol. 2017 Feb 21;17:49. doi: 10.1186/s12870-017-0992-8 (PMC5319169; doi:10.1186/s12870-017-0992-8)
Supplement: Supplementary file 1 — Supplementary material for “Comparative mitogenomics indicates respiratory competence in parasitic Viscum despite loss of complex I and extreme sequence divergence, and reveals horizontal gene transfer and remarkable variation in genome size”. Tables S1 to S2 and Figures S1 to S8. (PDF 12394 kb) [file 12870_2017_992_MOESM1_ESM.pdf]

Supplementary material for

Comparative mitogenomics indicates respiratory competence in parasitic *Viscum* despite loss of complex I and extreme sequence divergence, and reveals horizontal gene transfer and remarkable variation in genome size

Elizabeth Skippington, Todd J. Barkman, Danny W. Rice and Jeffrey D. Palmer*

*Corresponding author. E-mail: jpalmer@indiana.edu

This PDF file includes:

Tables S1 to S2
Figures S1 to S8

Table S1. Gene content of the *Viscum album* mitogenome.

Gene	Product	Coordinates	
atp1	ATPase subunit 1	439103	437565
atp4	ATPase subunit 4	509449	508976
atp6	ATPase subunit 6	99644	100558
atp8	ATPase subunit 8	52588	52094
atp9	ATPase subunit 9	497342	497118
ccmB	cytochrome c biogenesis subunit B	273822	274385
ccmC	cytochrome c biogenesis subunit C	418365	419021
ccmFc	cytochrome c biogenesis subunit FC	493580	492756
		491957	491475
ccmFn	cytochrome c biogenesis subunit FN	512059	510281
cob	apocytochrome b	95609	96820
cox1	cytochrome c oxidase subunit 1	51203	50493
		49500	48610
cox2	cytochrome c oxidase subunit 2	502063	501692
		43549	43860
		46039	46107
cox3	cytochrome c oxidase subunit 3	173779	174519
matR	maturase (splicing factor)	194269	192311
mttB	Tat protein translocator subunit 2	18014	18781
rpl16	ribosomal protein L16	518119	518496
rps3	ribosomal protein S3	516629	518086
rps4	ribosomal protein S4	413310	414317
rps10	ribosomal protein S10	423343	423068
rps12	ribosomal protein S12	177860	178279
sdh3	succinate dehydrogenase subunit 3	282757	282981
rrn5	5S ribosomal RNA	380044	380161
rrn18	18S ribosomal RNA	377450	379569
rrn26	26S ribosomal RNA	21049	25415
trnG-GCC	tRNA-Gly	92604	92533
trnK-TTT	tRNA-Lys	500844	500766
trnM-CAT	tRNA-Met	42653	42728
trnM-CAT	tRNA-Met	412252	412180
trnW-CCA	tRNA-Trp	6553	6628
trnW-CCA	tRNA-Trp	148987	149060

Table S2. Test for shifts in selective pressures using RELAX [1].

Locus	Selection intensity *k*	*P* Value
atp1	0.17	1.84E-07
atp6	0.20	4.06E-08
atp9	0.49	6.40E-05
ccmC	0.01	5.91E-05
cox1	0.27	3.81E-05
cox2	0.34	2.22E-04
cox3	0.22	1.07E-05
cob	0.70	2.66E-03
rps12	3.40	2.24E-02

Eight of the nine best conserved protein genes in *Viscum* showed a significant signature of relaxed selection ($k < 1$).

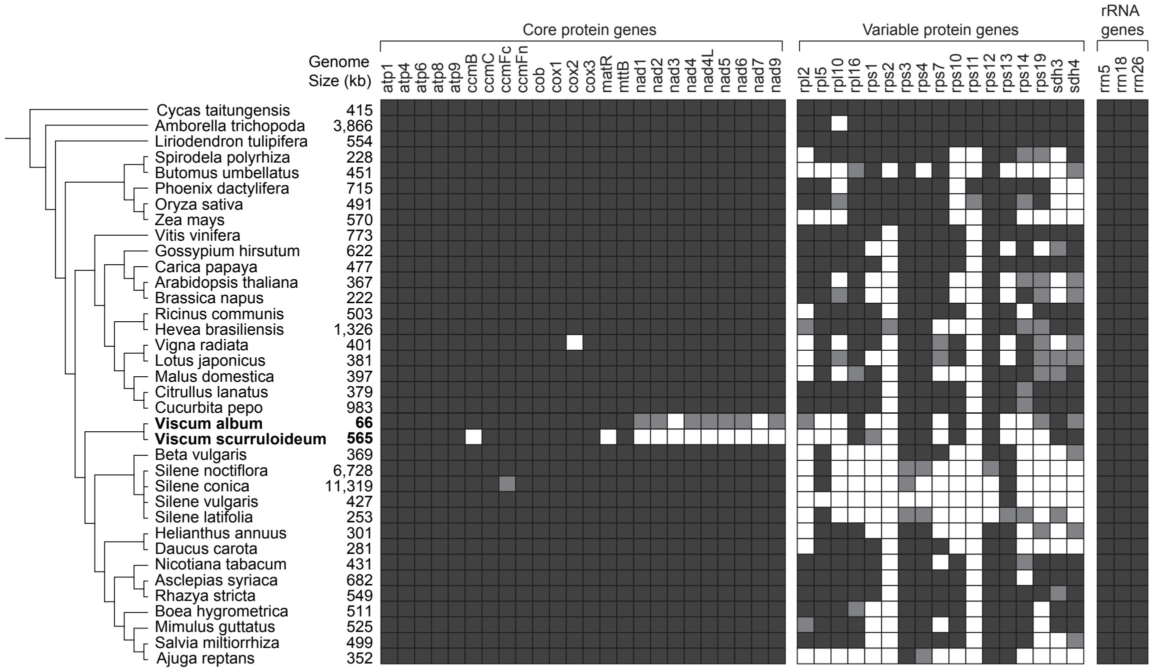

Fig. S1 Genome size and protein- and rRNA-gene content of 35 sequenced angiosperm mitochondrial genomes and outgroup *Cycas taitungensis*. Intact genes are dark gray, pseudogenes are light gray, and absent genes are white.

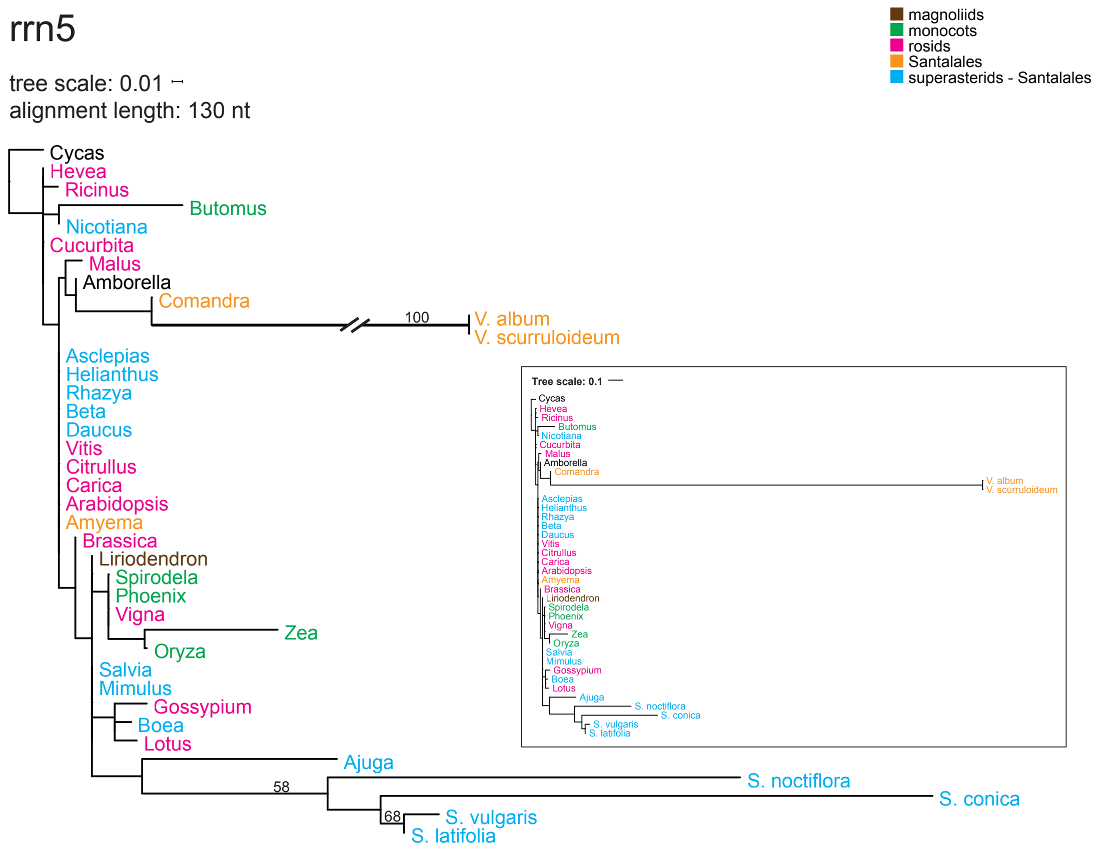

Fig. S2 Maximum likelihood phylogenies based on all-position nucleotide alignments for three mitochondrial rRNA genes and 21 mitochondrial protein genes. Bootstrap support values > 50% from 1,000 replicates are shown. For most genes, one or a few branch lengths (usually only the branch leading to *Viscum*) are shown at a reduced scale in the main tree, with all branches shown to the same scale in the accompanying inset tree.

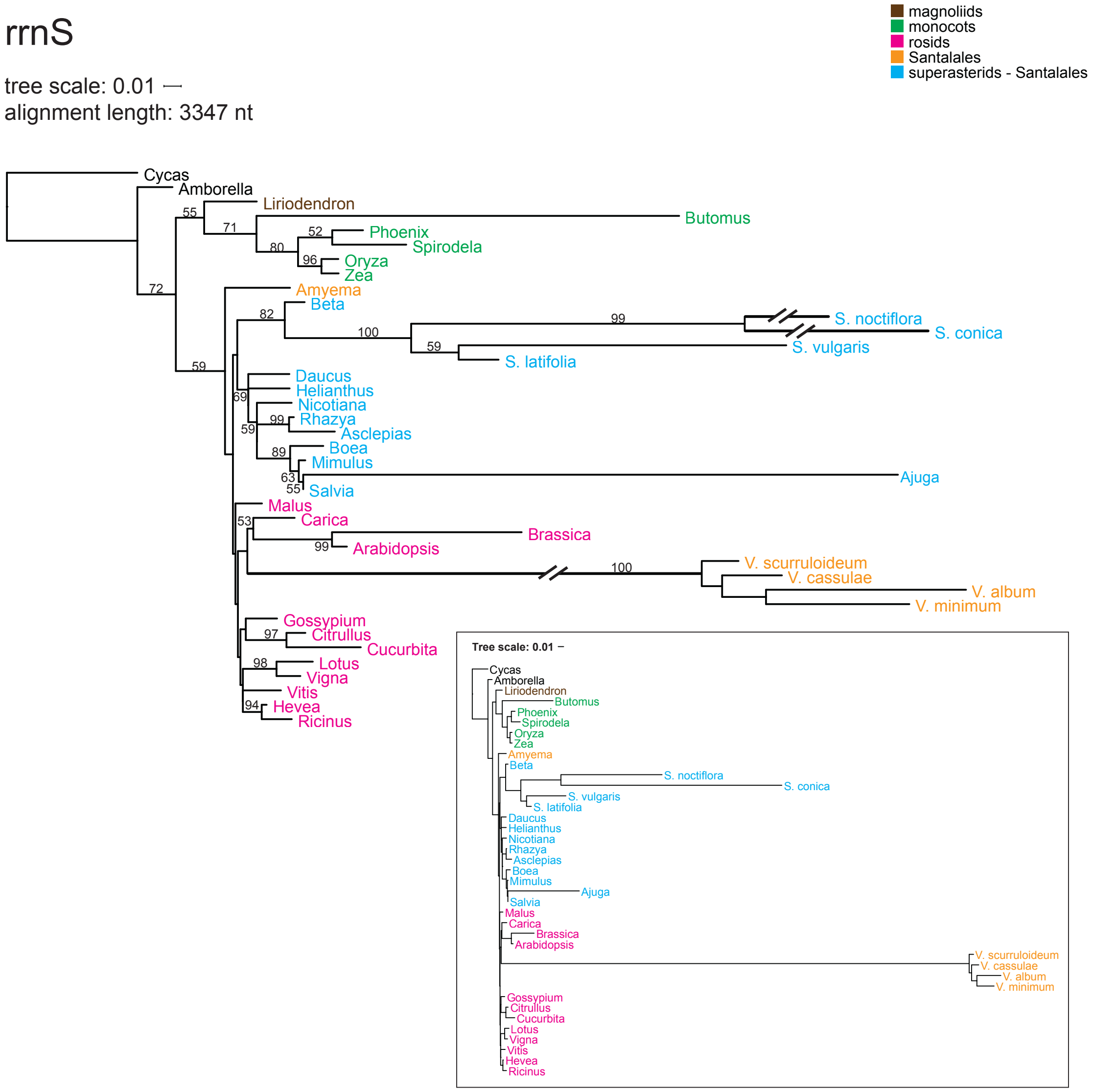

Fig. S2 Maximum likelihood phylogenies based on all-position nucleotide alignments for three mitochondrial rRNA genes and 21 mitochondrial protein genes. Bootstrap support values > 50% from 1,000 replicates are shown. For most genes, one or a few branch lengths (usually only the branch leading to *Viscum*) are shown at a reduced scale in the main tree, with all branches shown to the same scale in the accompanying inset tree.

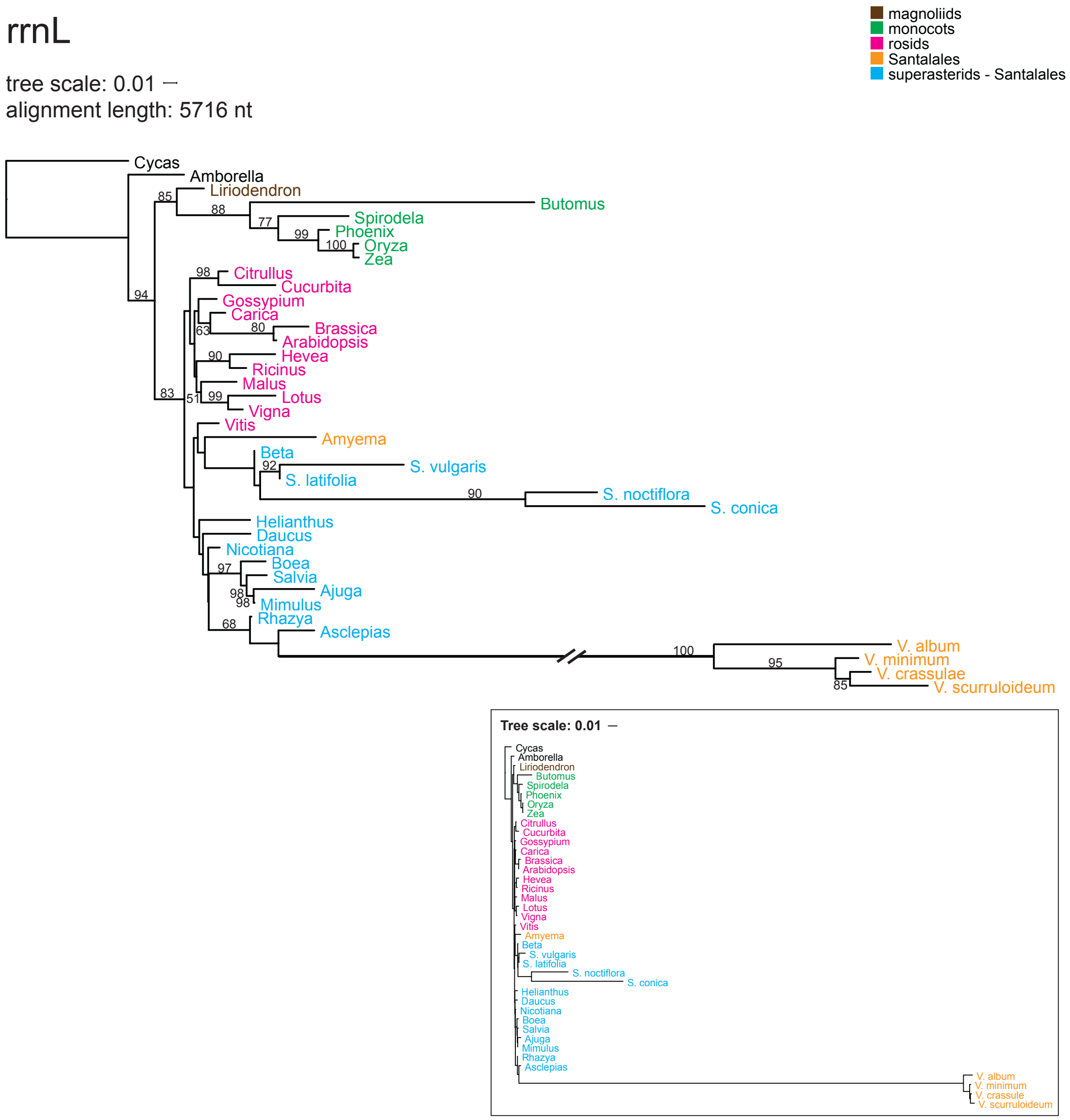

Fig. S2 Maximum likelihood phylogenies based on all-position nucleotide alignments for three mitochondrial rRNA genes and 21 mitochondrial protein genes. Bootstrap support values > 50% from 1,000 replicates are shown. For most genes, one or a few branch lengths (usually only the branch leading to *Viscum*) are shown at a reduced scale in the main tree, with all branches shown to the same scale in the accompanying inset tree.

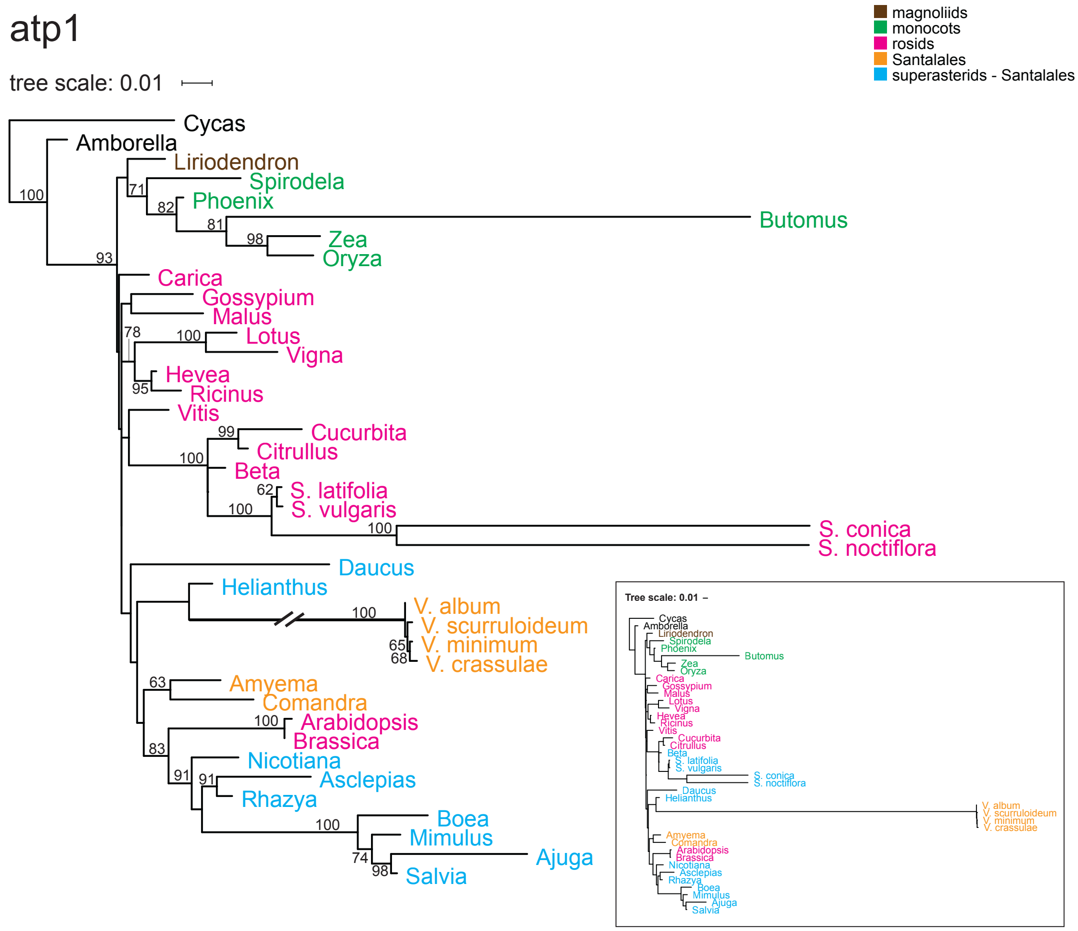

Fig. S2 Maximum likelihood phylogenies based on all-position nucleotide alignments for three mitochondrial rRNA genes and 21 mitochondrial protein genes. Bootstrap support values > 50% from 1,000 replicates are shown. For most genes, one or a few branch lengths (usually only the branch leading to *Viscum*) are shown at a reduced scale in the main tree, with all branches shown to the same scale in the accompanying inset tree.

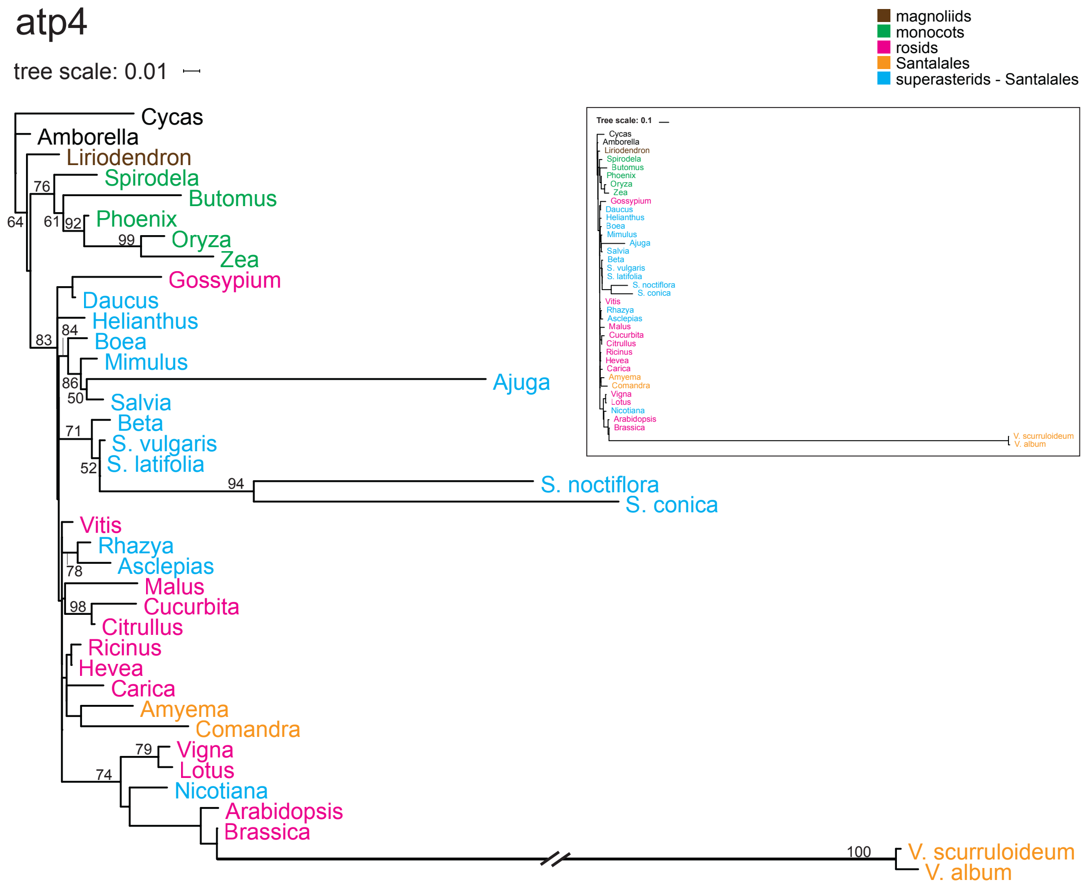

Fig. S2 Maximum likelihood phylogenies based on all-position nucleotide alignments for three mitochondrial rRNA genes and 21 mitochondrial protein genes. Bootstrap support values > 50% from 1,000 replicates are shown. For most genes, one or a few branch lengths (usually only the branch leading to *Viscum*) are shown at a reduced scale in the main tree, with all branches shown to the same scale in the accompanying inset tree.

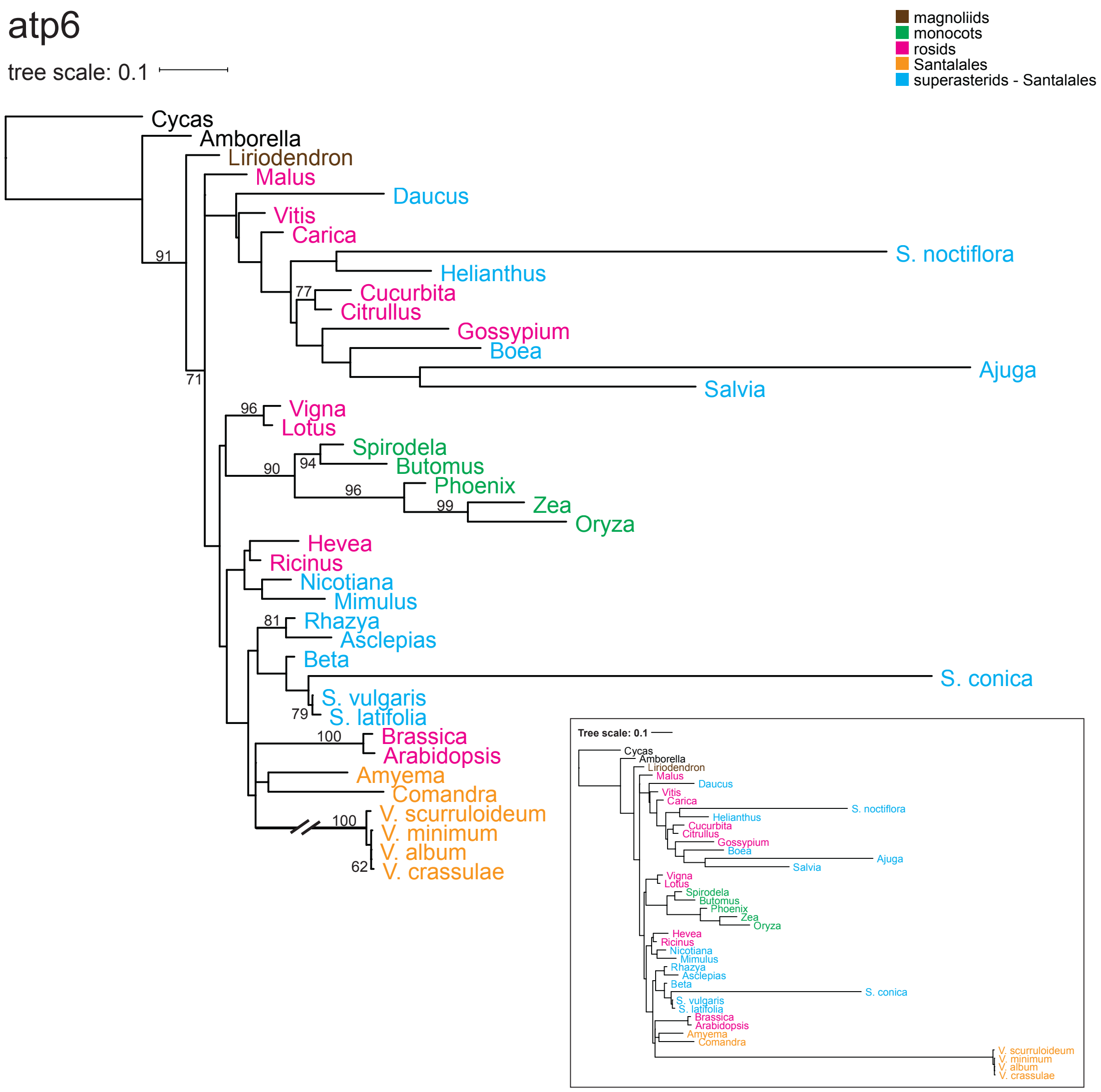

Fig. S2 Maximum likelihood phylogenies based on all-position nucleotide alignments for three mitochondrial rRNA genes and 21 mitochondrial protein genes. Bootstrap support values > 50% from 1,000 replicates are shown. For most genes, one or a few branch lengths (usually only the branch leading to *Viscum*) are shown at a reduced scale in the main tree, with all branches shown to the same scale in the accompanying inset tree.

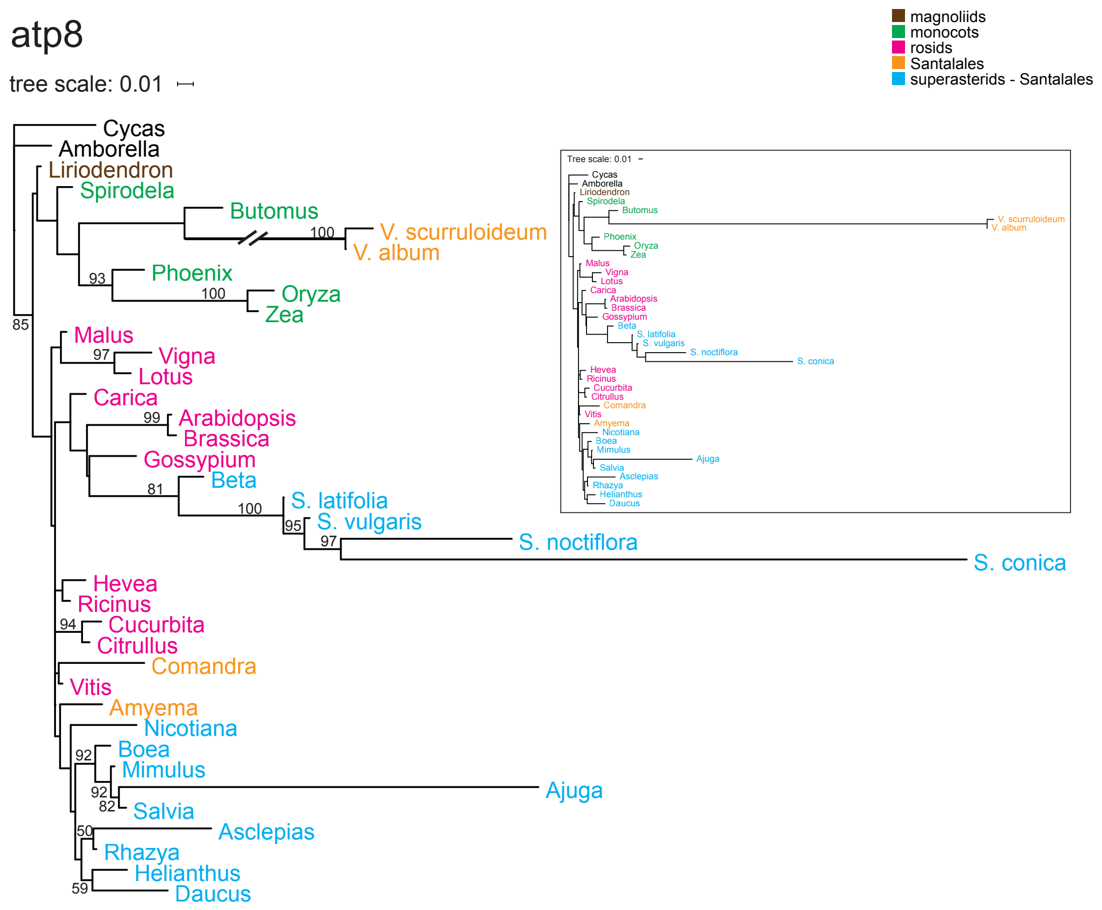

Fig. S2 Maximum likelihood phylogenies based on all-position nucleotide alignments for three mitochondrial rRNA genes and 21 mitochondrial protein genes. Bootstrap support values > 50% from 1,000 replicates are shown. For most genes, one or a few branch lengths (usually only the branch leading to *Viscum*) are shown at a reduced scale in the main tree, with all branches shown to the same scale in the accompanying inset tree.

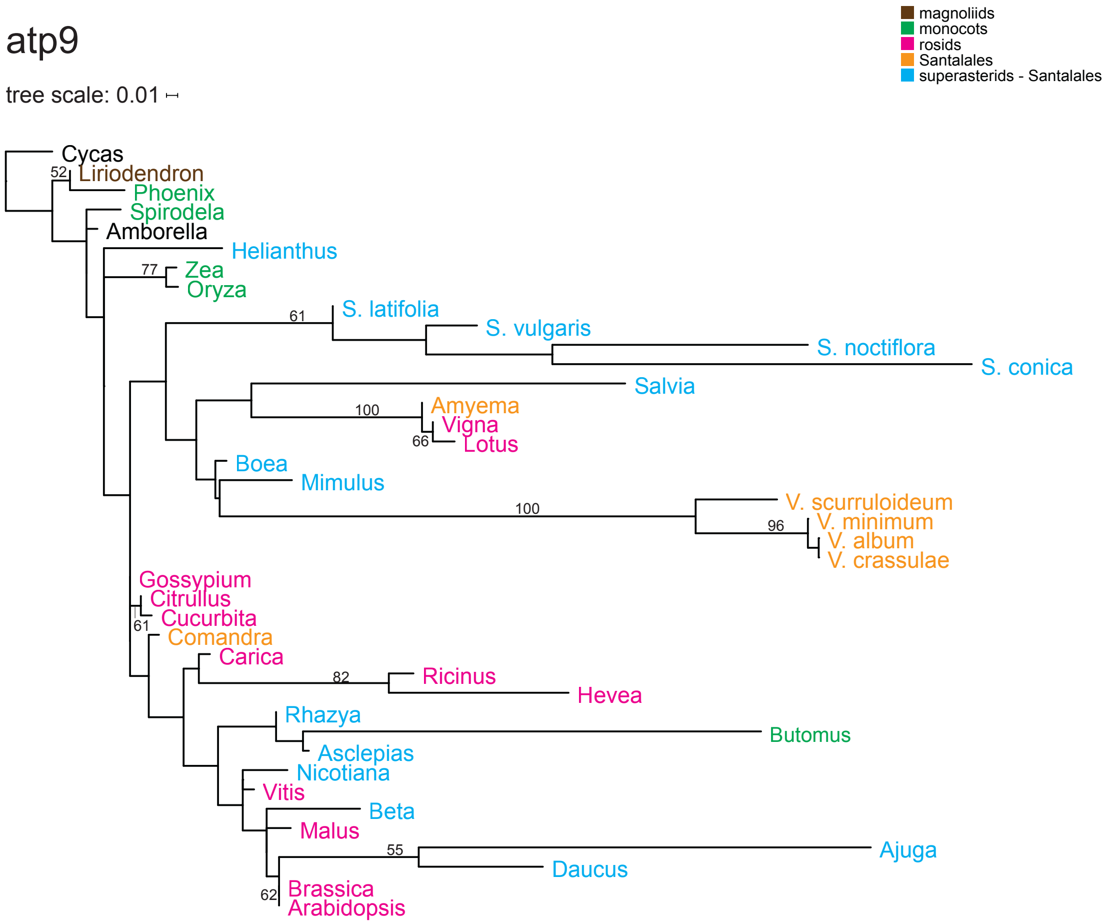

Fig. S2 Maximum likelihood phylogenies based on all-position nucleotide alignments for three mitochondrial rRNA genes and 21 mitochondrial protein genes. Bootstrap support values > 50% from 1,000 replicates are shown. For most genes, one or a few branch lengths (usually only the branch leading to *Viscum*) are shown at a reduced scale in the main tree, with all branches shown to the same scale in the accompanying inset tree.

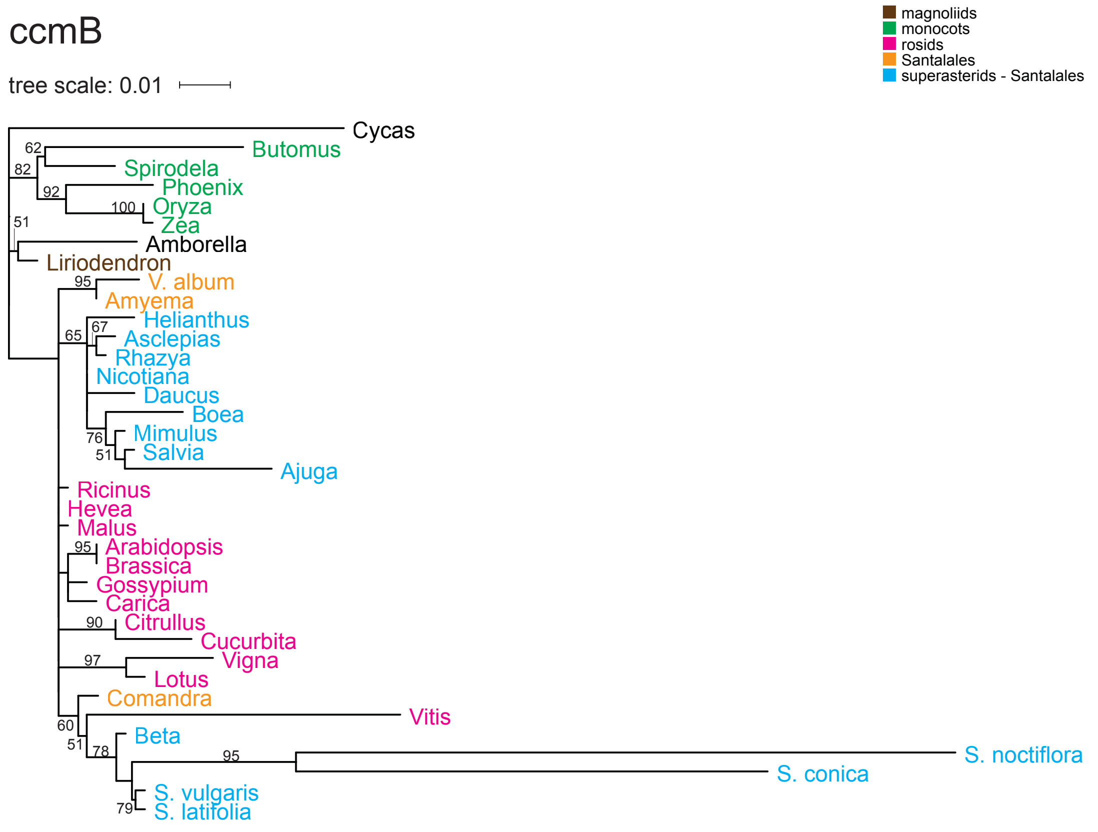

Fig. S2 Maximum likelihood phylogenies based on all-position nucleotide alignments for three mitochondrial rRNA genes and 21 mitochondrial protein genes. Bootstrap support values > 50% from 1,000 replicates are shown. For most genes, one or a few branch lengths (usually only the branch leading to *Viscum*) are shown at a reduced scale in the main tree, with all branches shown to the same scale in the accompanying inset tree.

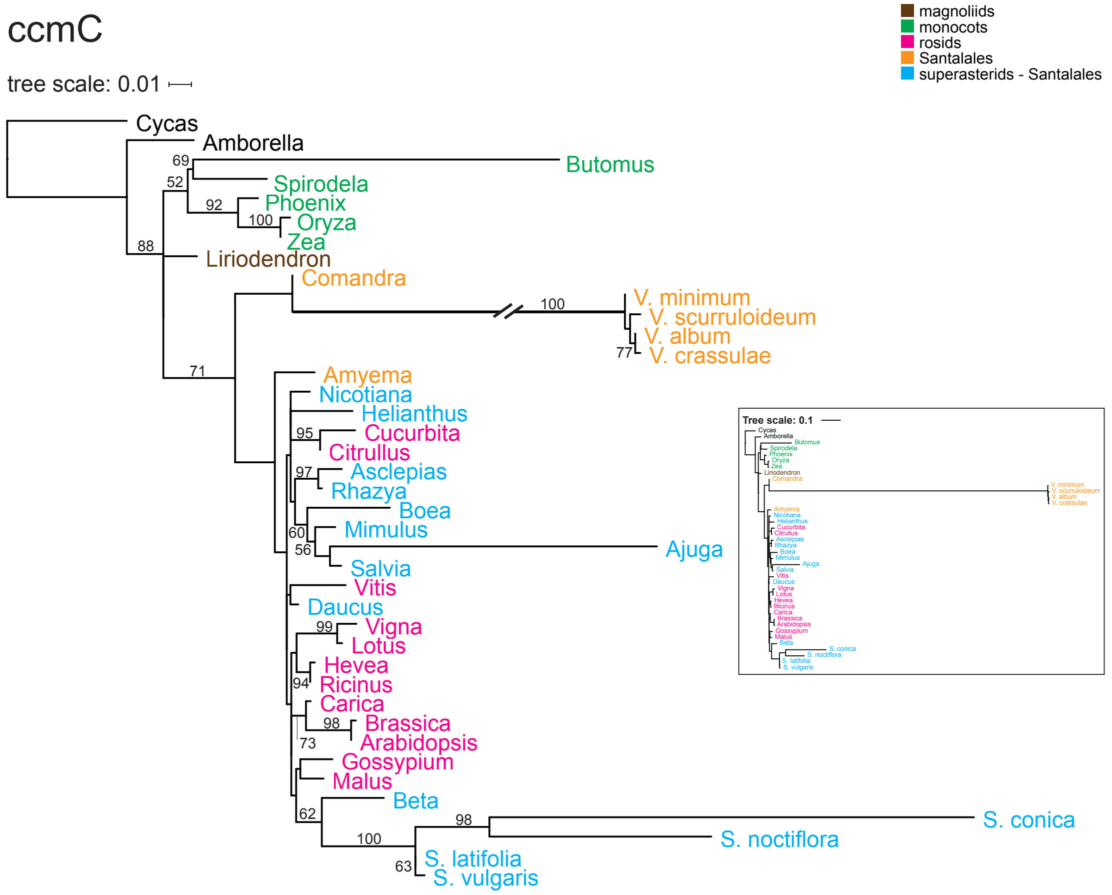

Fig. S2 Maximum likelihood phylogenies based on all-position nucleotide alignments for three mitochondrial rRNA genes and 21 mitochondrial protein genes. Bootstrap support values > 50% from 1,000 replicates are shown. For most genes, one or a few branch lengths (usually only the branch leading to *Viscum*) are shown at a reduced scale in the main tree, with all branches shown to the same scale in the accompanying inset tree.

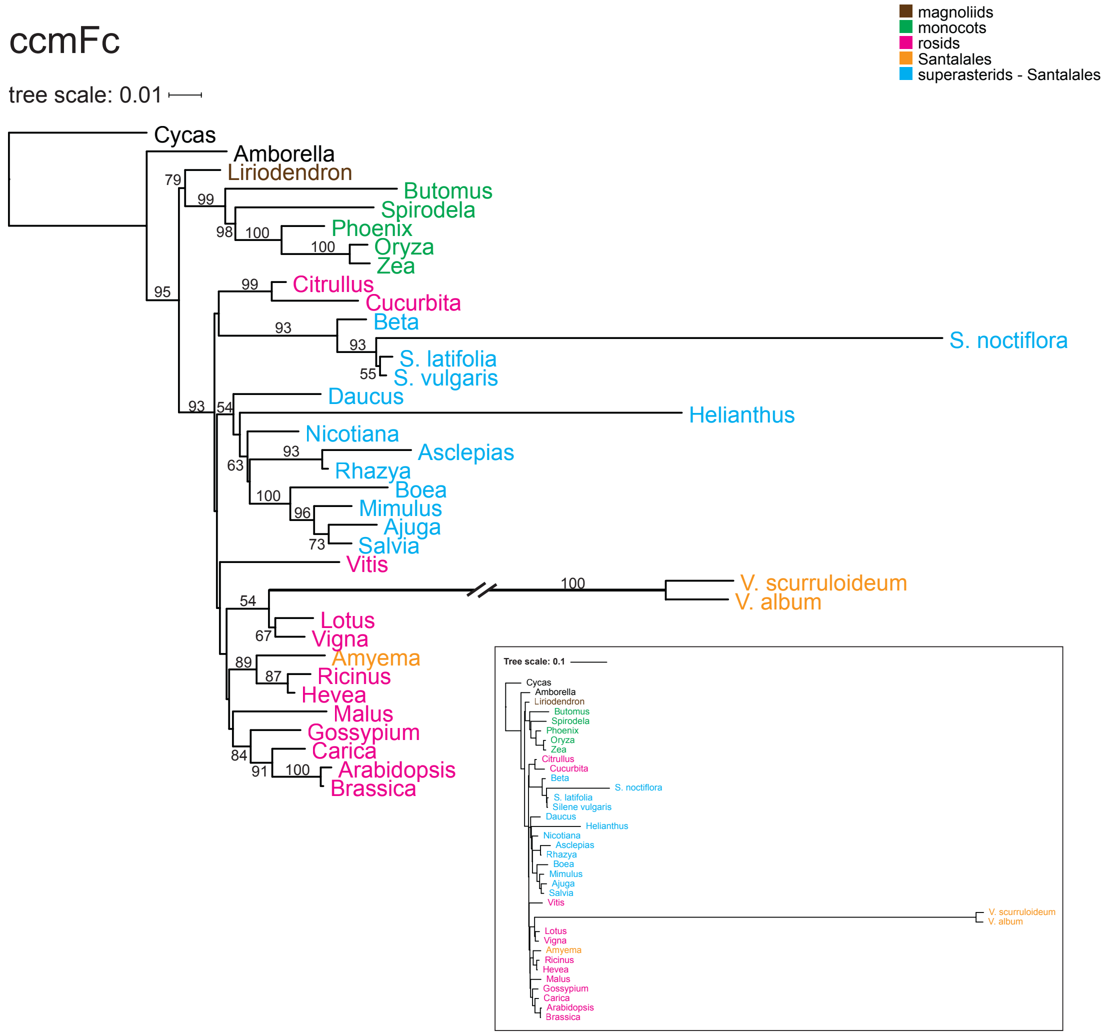

Fig. S2 Maximum likelihood phylogenies based on all-position nucleotide alignments for three mitochondrial rRNA genes and 21 mitochondrial protein genes. Bootstrap support values > 50% from 1,000 replicates are shown. For most genes, one or a few branch lengths (usually only the branch leading to *Viscum*) are shown at a reduced scale in the main tree, with all branches shown to the same scale in the accompanying inset tree.

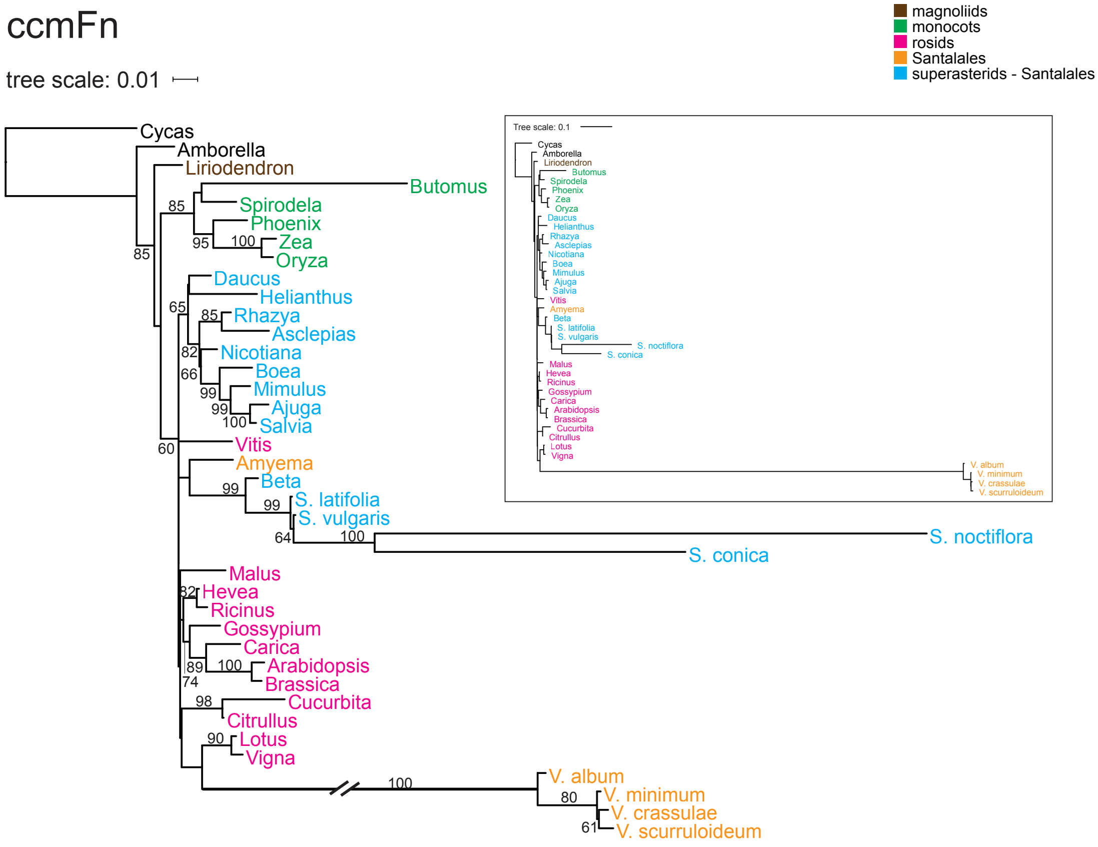

Fig. S2 Maximum likelihood phylogenies based on all-position nucleotide alignments for three mitochondrial rRNA genes and 21 mitochondrial protein genes. Bootstrap support values > 50% from 1,000 replicates are shown. For most genes, one or a few branch lengths (usually only the branch leading to *Viscum*) are shown at a reduced scale in the main tree, with all branches shown to the same scale in the accompanying inset tree.

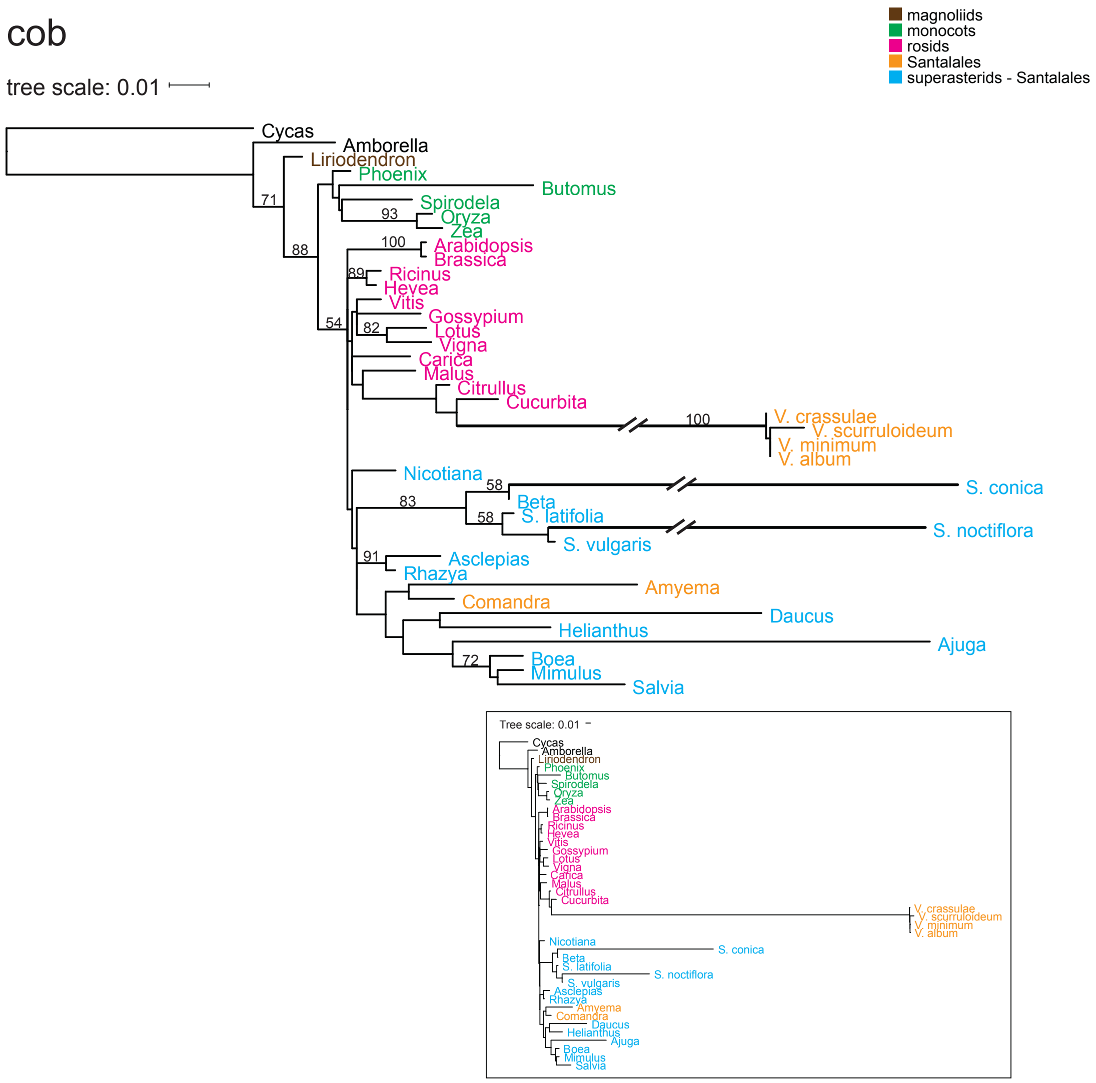

Fig. S2 Maximum likelihood phylogenies based on all-position nucleotide alignments for three mitochondrial rRNA genes and 21 mitochondrial protein genes. Bootstrap support values > 50% from 1,000 replicates are shown. For most genes, one or a few branch lengths (usually only the branch leading to *Viscum*) are shown at a reduced scale in the main tree, with all branches shown to the same scale in the accompanying inset tree.

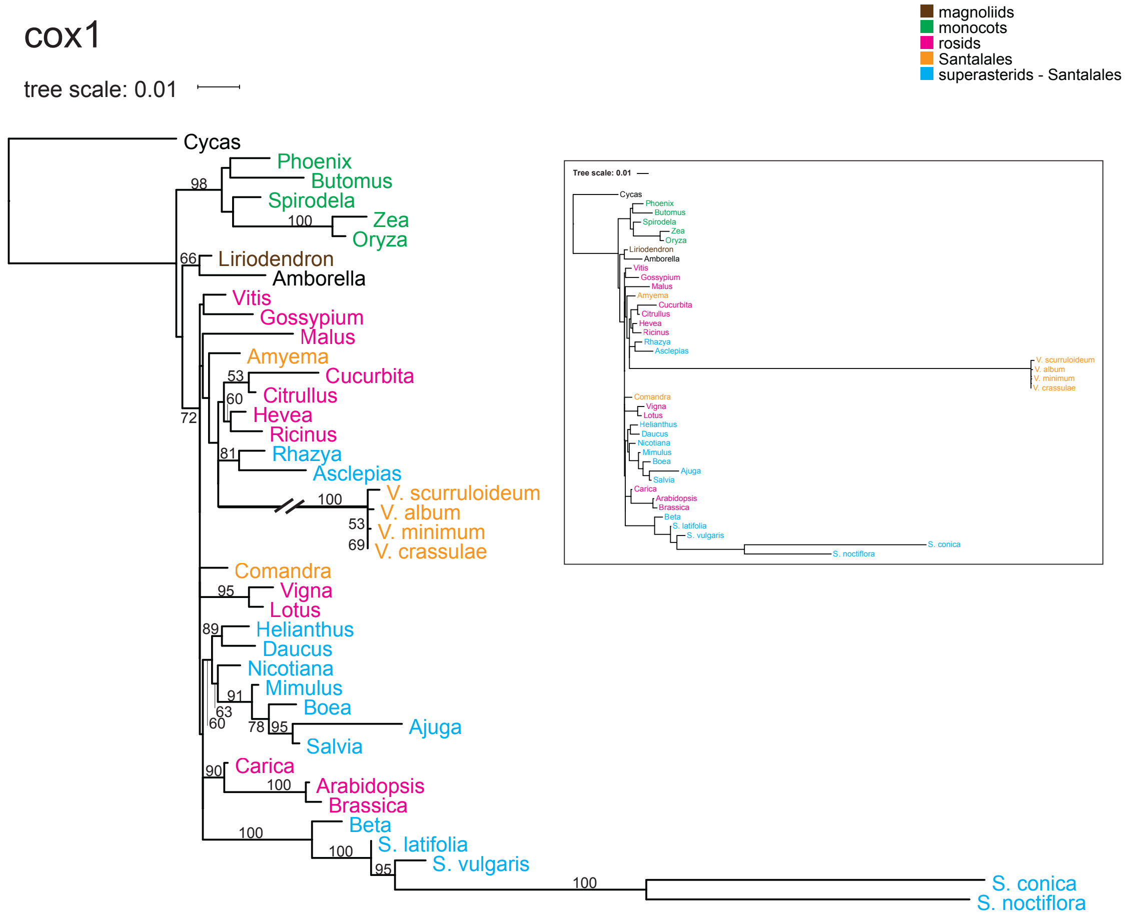

Fig. S2 Maximum likelihood phylogenies based on all-position nucleotide alignments for three mitochondrial rRNA genes and 21 mitochondrial protein genes. Bootstrap support values > 50% from 1,000 replicates are shown. For most genes, one or a few branch lengths (usually only the branch leading to *Viscum*) are shown at a reduced scale in the main tree, with all branches shown to the same scale in the accompanying inset tree.

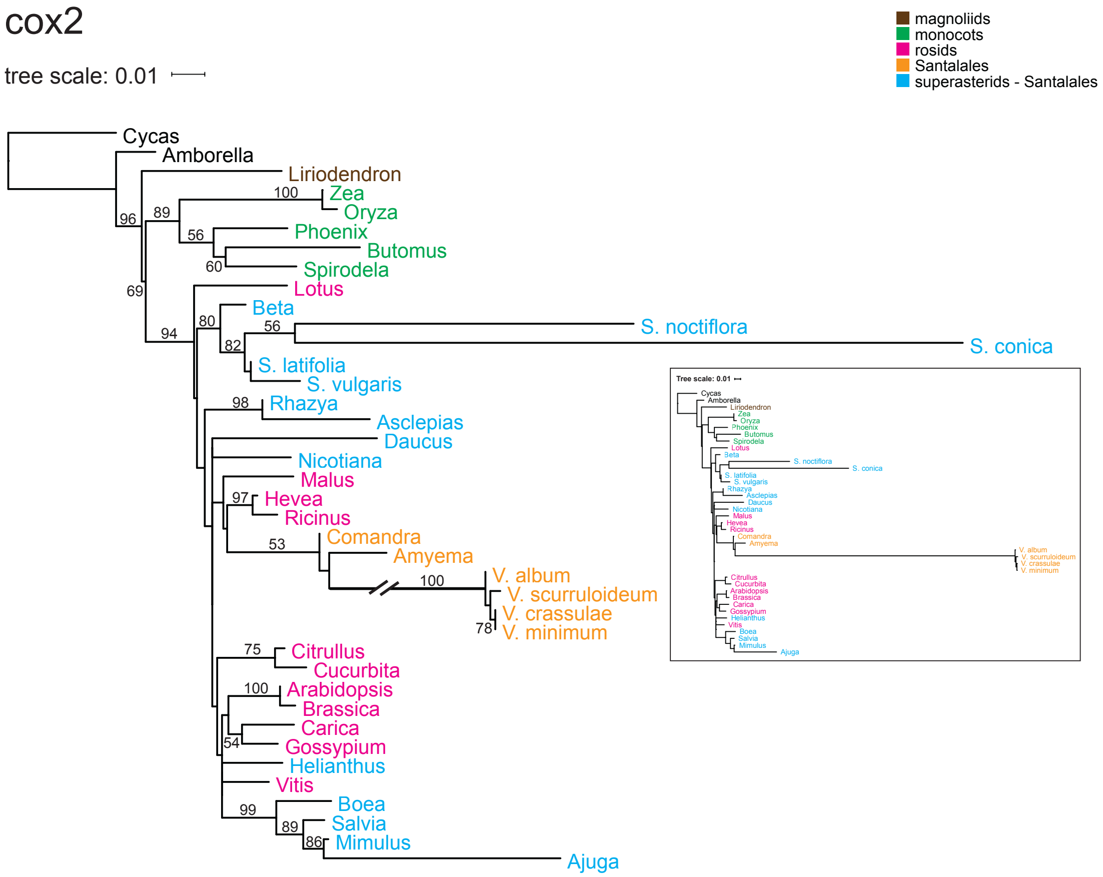

Fig. S2 Maximum likelihood phylogenies based on all-position nucleotide alignments for three mitochondrial rRNA genes and 21 mitochondrial protein genes. Bootstrap support values > 50% from 1,000 replicates are shown. For most genes, one or a few branch lengths (usually only the branch leading to *Viscum*) are shown at a reduced scale in the main tree, with all branches shown to the same scale in the accompanying inset tree.

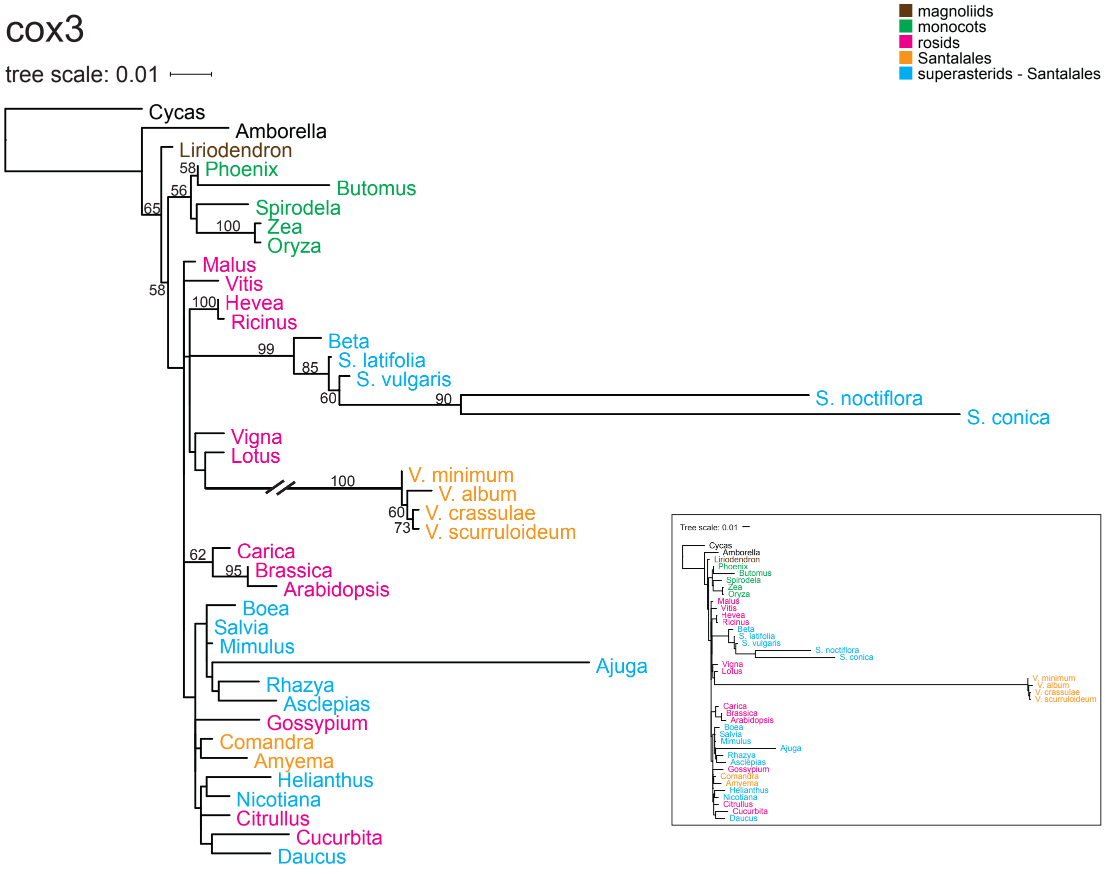

Fig. S2 Maximum likelihood phylogenies based on all-position nucleotide alignments for three mitochondrial rRNA genes and 21 mitochondrial protein genes. Bootstrap support values > 50% from 1,000 replicates are shown. For most genes, one or a few branch lengths (usually only the branch leading to *Viscum*) are shown at a reduced scale in the main tree, with all branches shown to the same scale in the accompanying inset tree.

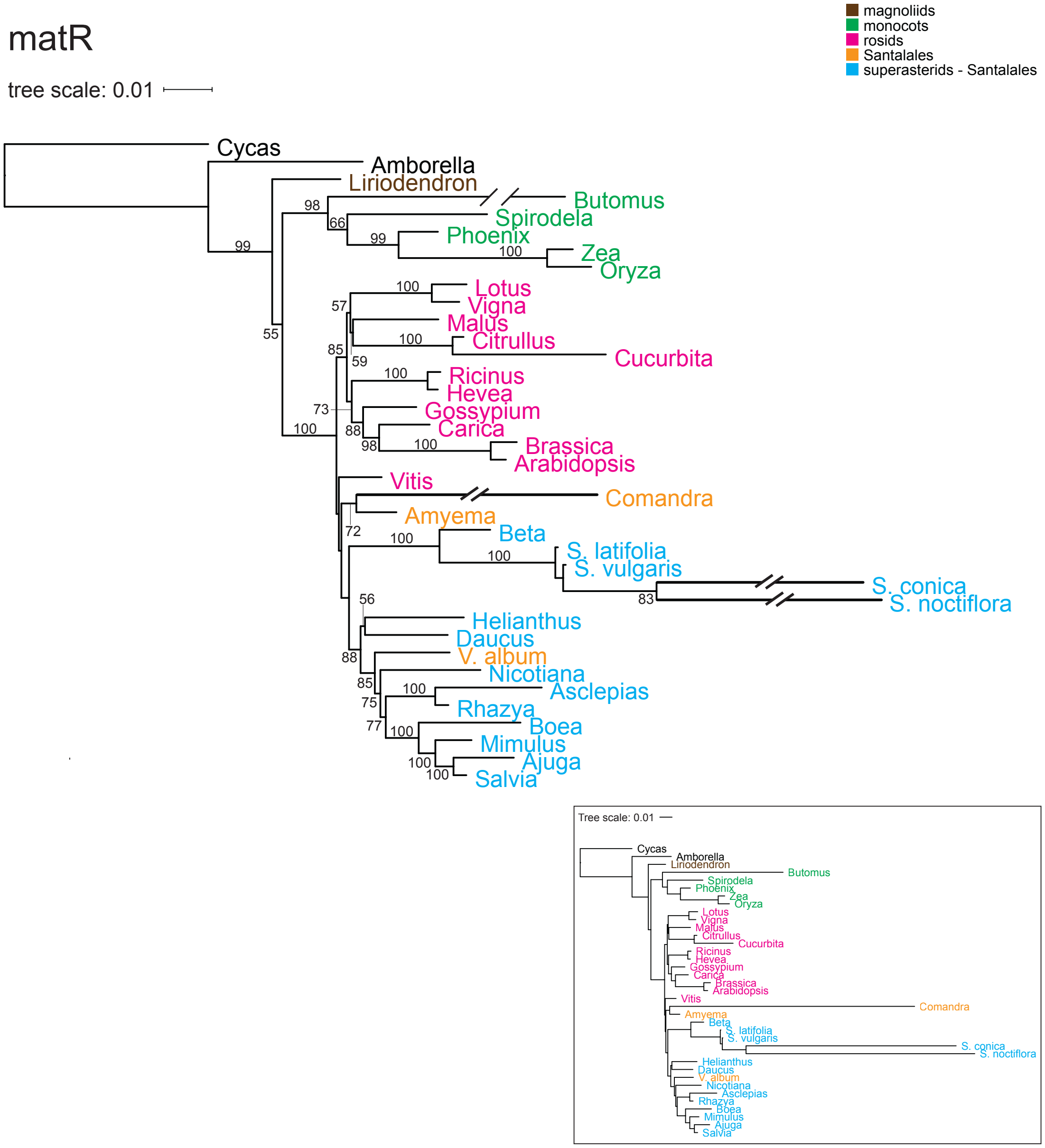

Fig. S2 Maximum likelihood phylogenies based on all-position nucleotide alignments for three mitochondrial rRNA genes and 21 mitochondrial protein genes. Bootstrap support values > 50% from 1,000 replicates are shown. For most genes, one or a few branch lengths (usually only the branch leading to *Viscum*) are shown at a reduced scale in the main tree, with all branches shown to the same scale in the accompanying inset tree.

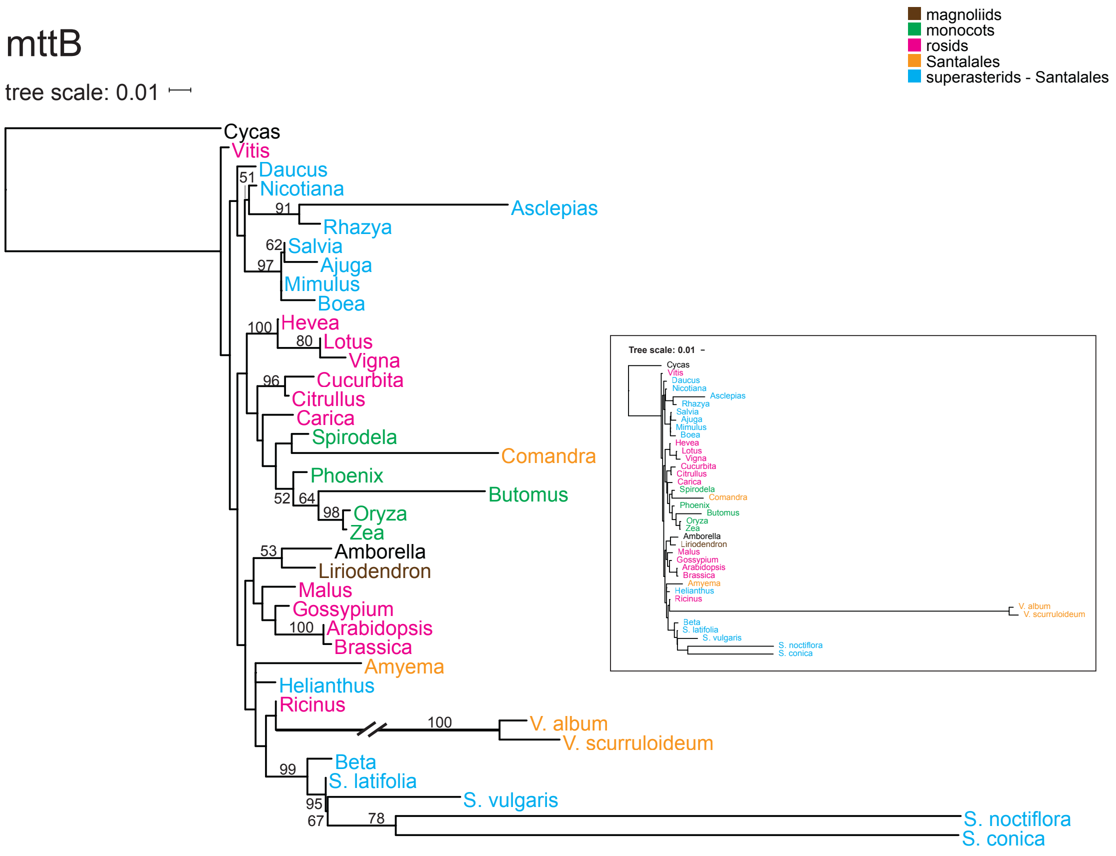

Fig. S2 Maximum likelihood phylogenies based on all-position nucleotide alignments for three mitochondrial rRNA genes and 21 mitochondrial protein genes. Bootstrap support values > 50% from 1,000 replicates are shown. For most genes, one or a few branch lengths (usually only the branch leading to *Viscum*) are shown at a reduced scale in the main tree, with all branches shown to the same scale in the accompanying inset tree.

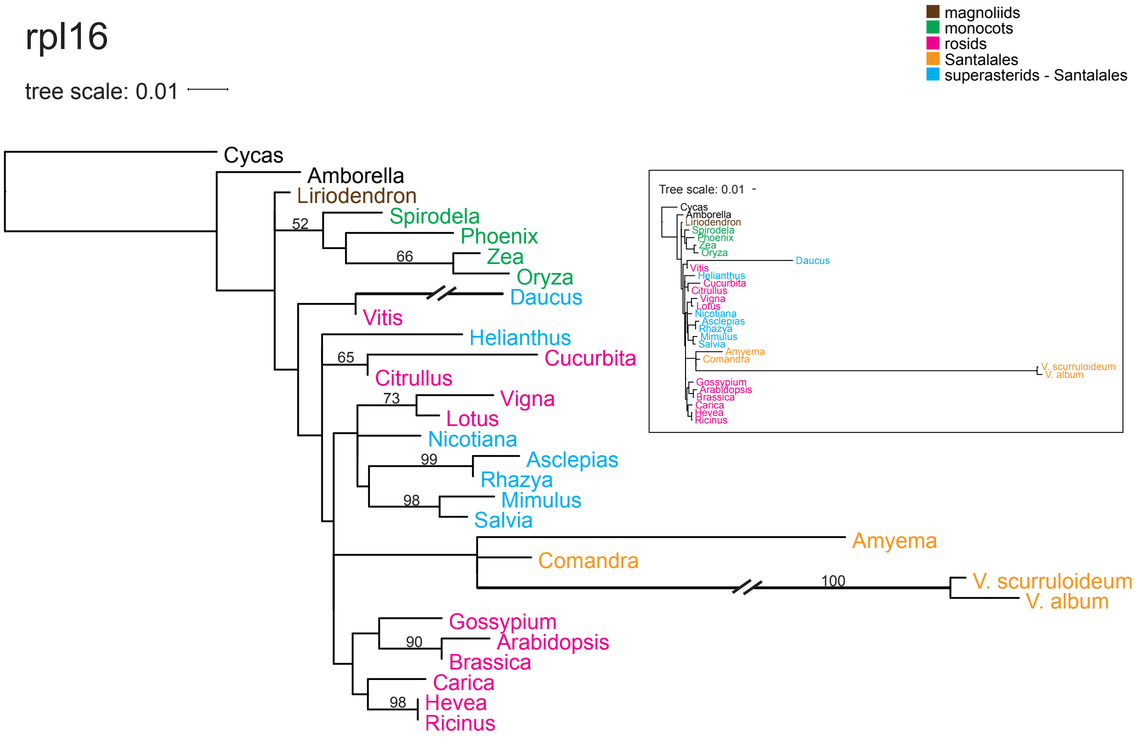

Fig. S2 Maximum likelihood phylogenies based on all-position nucleotide alignments for three mitochondrial rRNA genes and 21 mitochondrial protein genes. Bootstrap support values > 50% from 1,000 replicates are shown. For most genes, one or a few branch lengths (usually only the branch leading to *Viscum*) are shown at a reduced scale in the main tree, with all branches shown to the same scale in the accompanying inset tree.

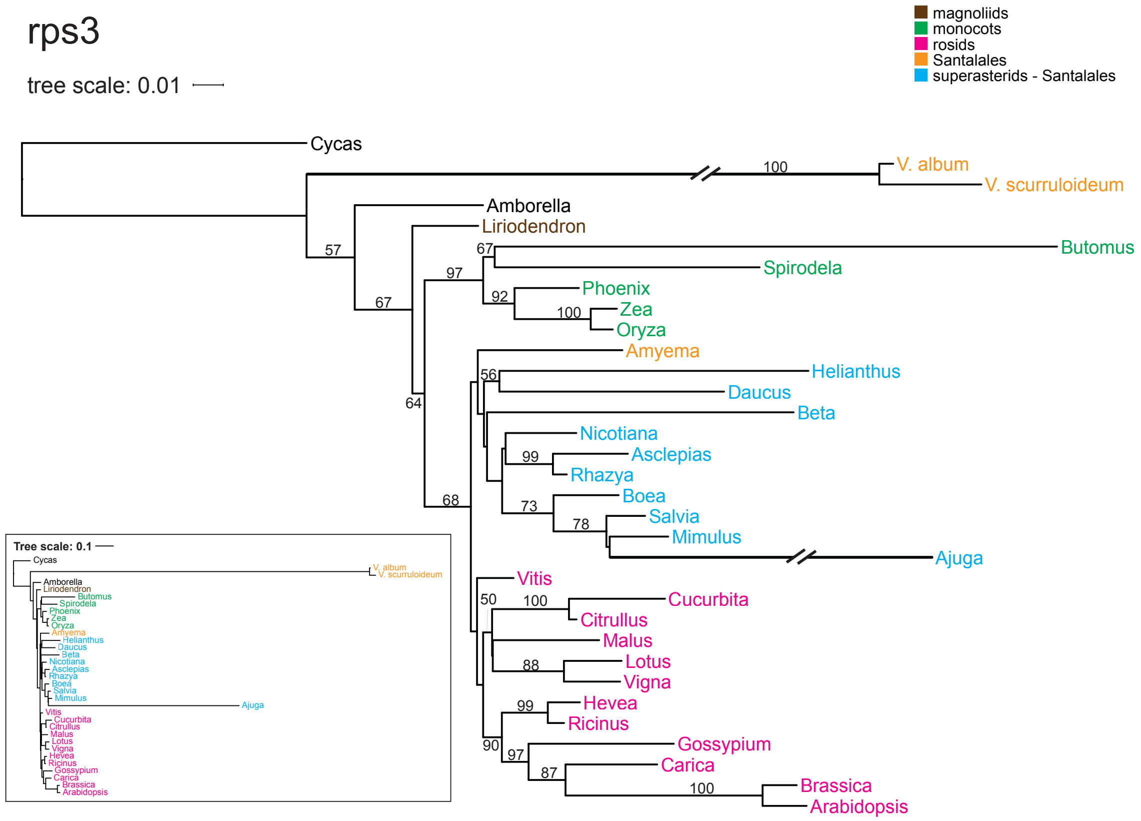

Fig. S2 Maximum likelihood phylogenies based on all-position nucleotide alignments for three mitochondrial rRNA genes and 21 mitochondrial protein genes. Bootstrap support values > 50% from 1,000 replicates are shown. For most genes, one or a few branch lengths (usually only the branch leading to *Viscum*) are shown at a reduced scale in the main tree, with all branches shown to the same scale in the accompanying inset tree.

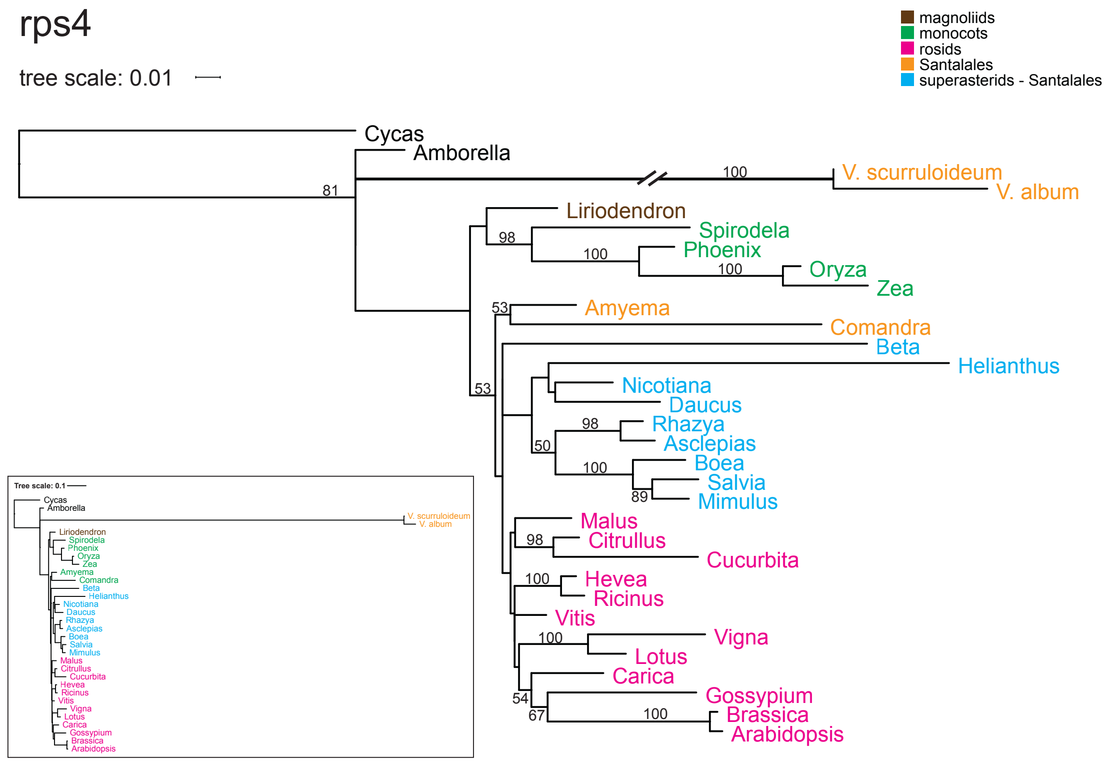

Fig. S2 Maximum likelihood phylogenies based on all-position nucleotide alignments for three mitochondrial rRNA genes and 21 mitochondrial protein genes. Bootstrap support values > 50% from 1,000 replicates are shown. For most genes, one or a few branch lengths (usually only the branch leading to *Viscum*) are shown at a reduced scale in the main tree, with all branches shown to the same scale in the accompanying inset tree.

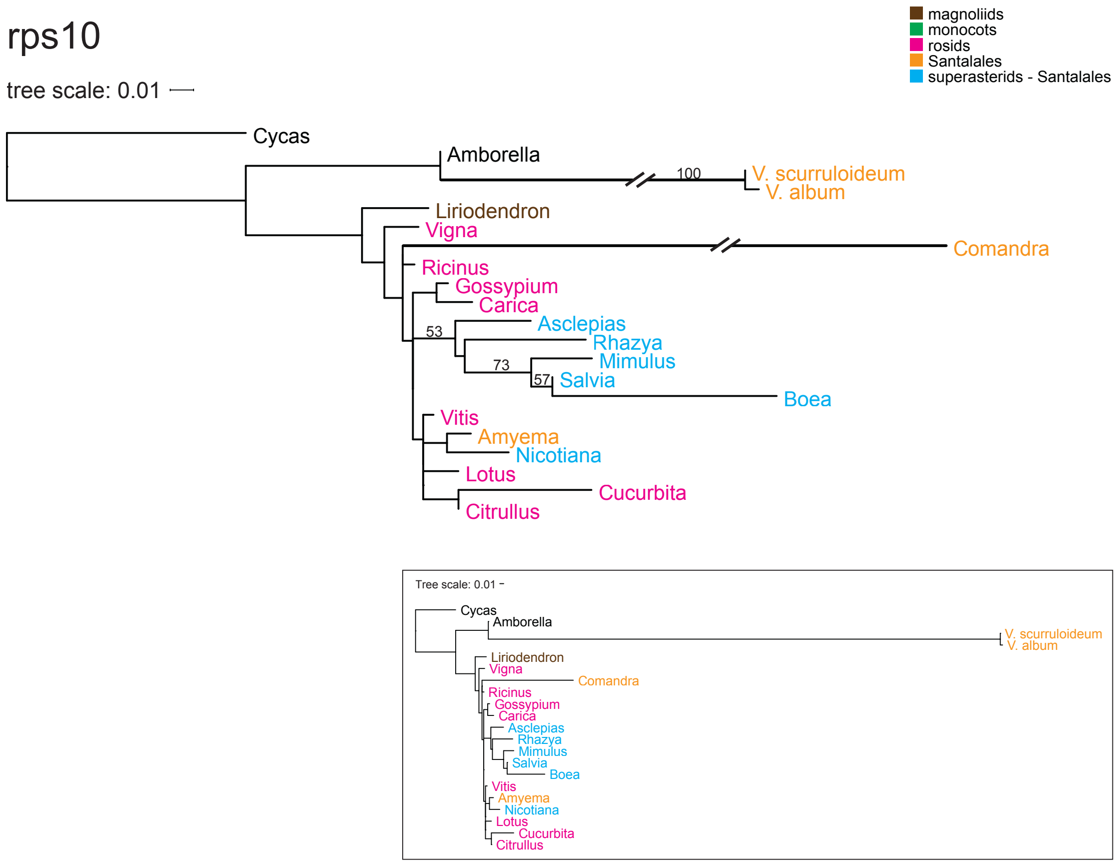

Fig. S2 Maximum likelihood phylogenies based on all-position nucleotide alignments for three mitochondrial rRNA genes and 21 mitochondrial protein genes. Bootstrap support values > 50% from 1,000 replicates are shown. For most genes, one or a few branch lengths (usually only the branch leading to *Viscum*) are shown at a reduced scale in the main tree, with all branches shown to the same scale in the accompanying inset tree.

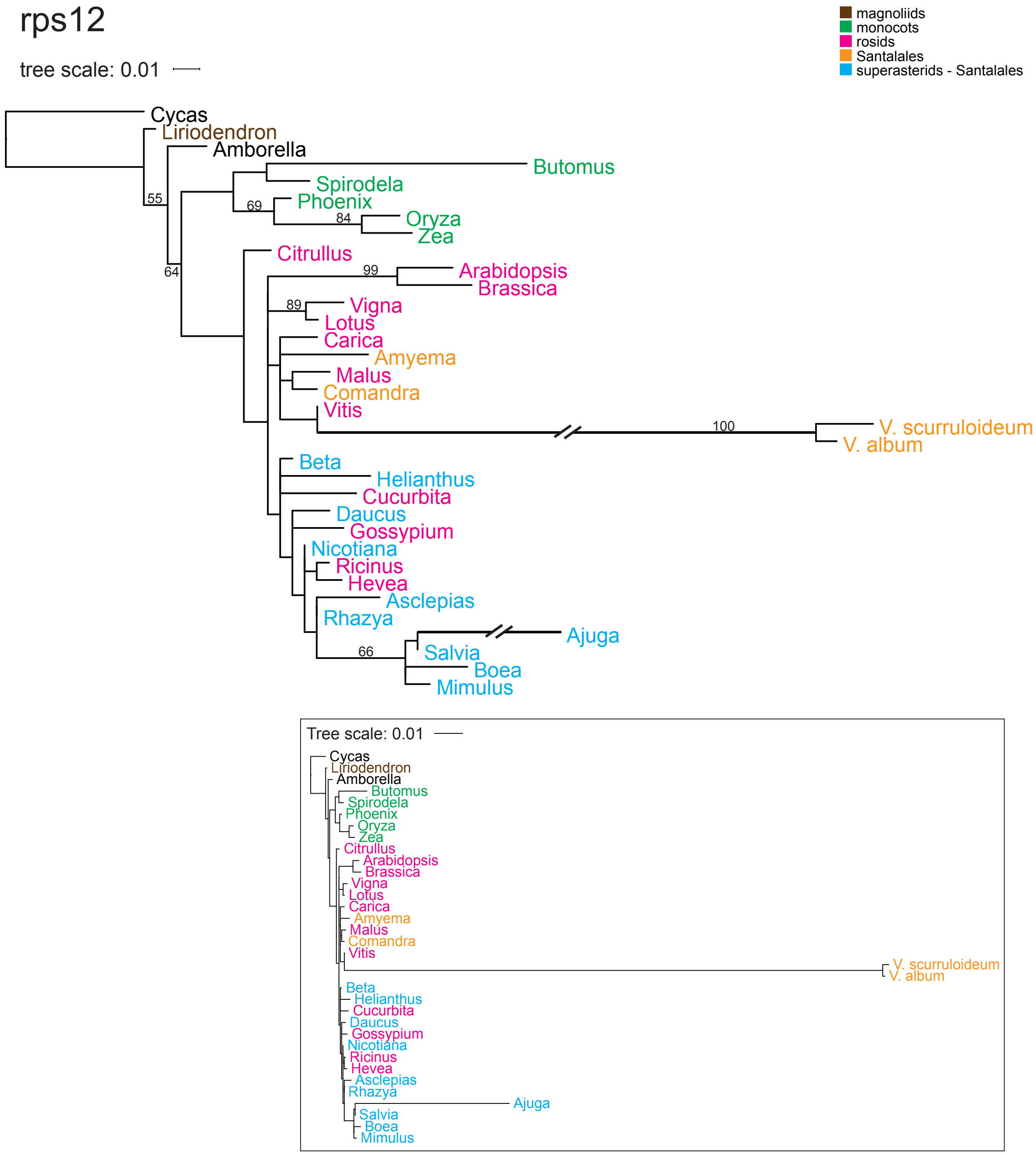

Fig. S2 Maximum likelihood phylogenies based on all-position nucleotide alignments for three mitochondrial rRNA genes and 21 mitochondrial protein genes. Bootstrap support values > 50% from 1,000 replicates are shown. For most genes, one or a few branch lengths (usually only the branch leading to *Viscum*) are shown at a reduced scale in the main tree, with all branches shown to the same scale in the accompanying inset tree.

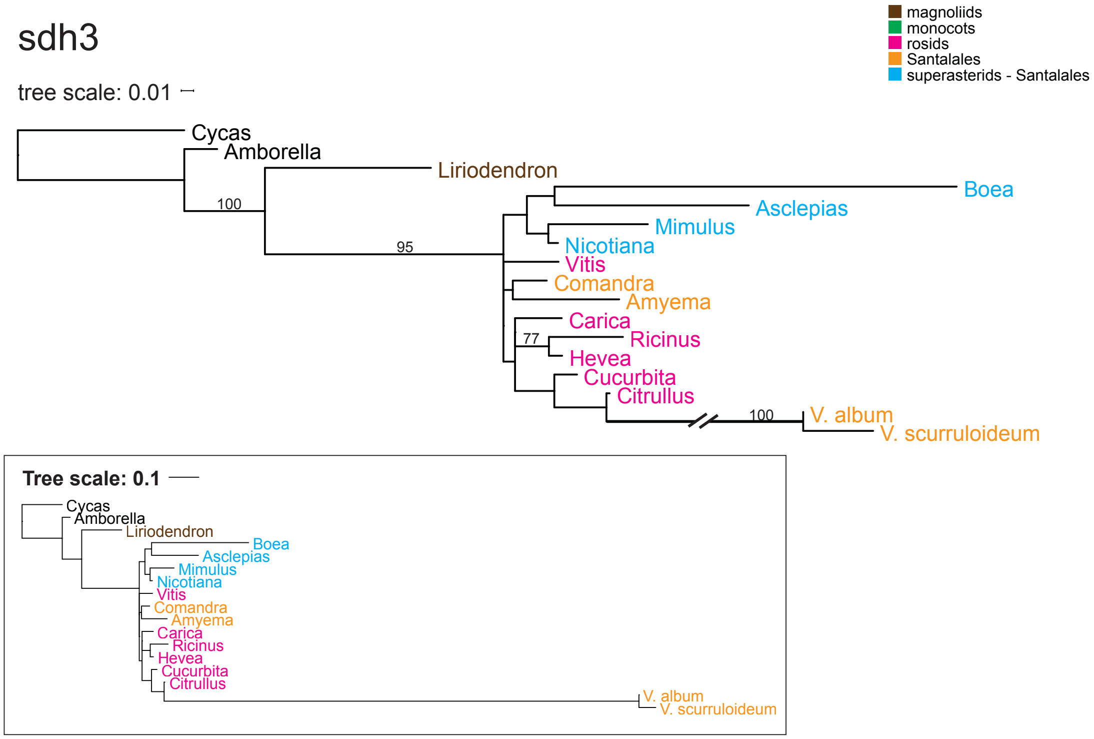

Fig. S2 Maximum likelihood phylogenies based on all-position nucleotide alignments for three mitochondrial rRNA genes and 21 mitochondrial protein genes. Bootstrap support values > 50% from 1,000 replicates are shown. For most genes, one or a few branch lengths (usually only the branch leading to *Viscum*) are shown at a reduced scale in the main tree, with all branches shown to the same scale in the accompanying inset tree.

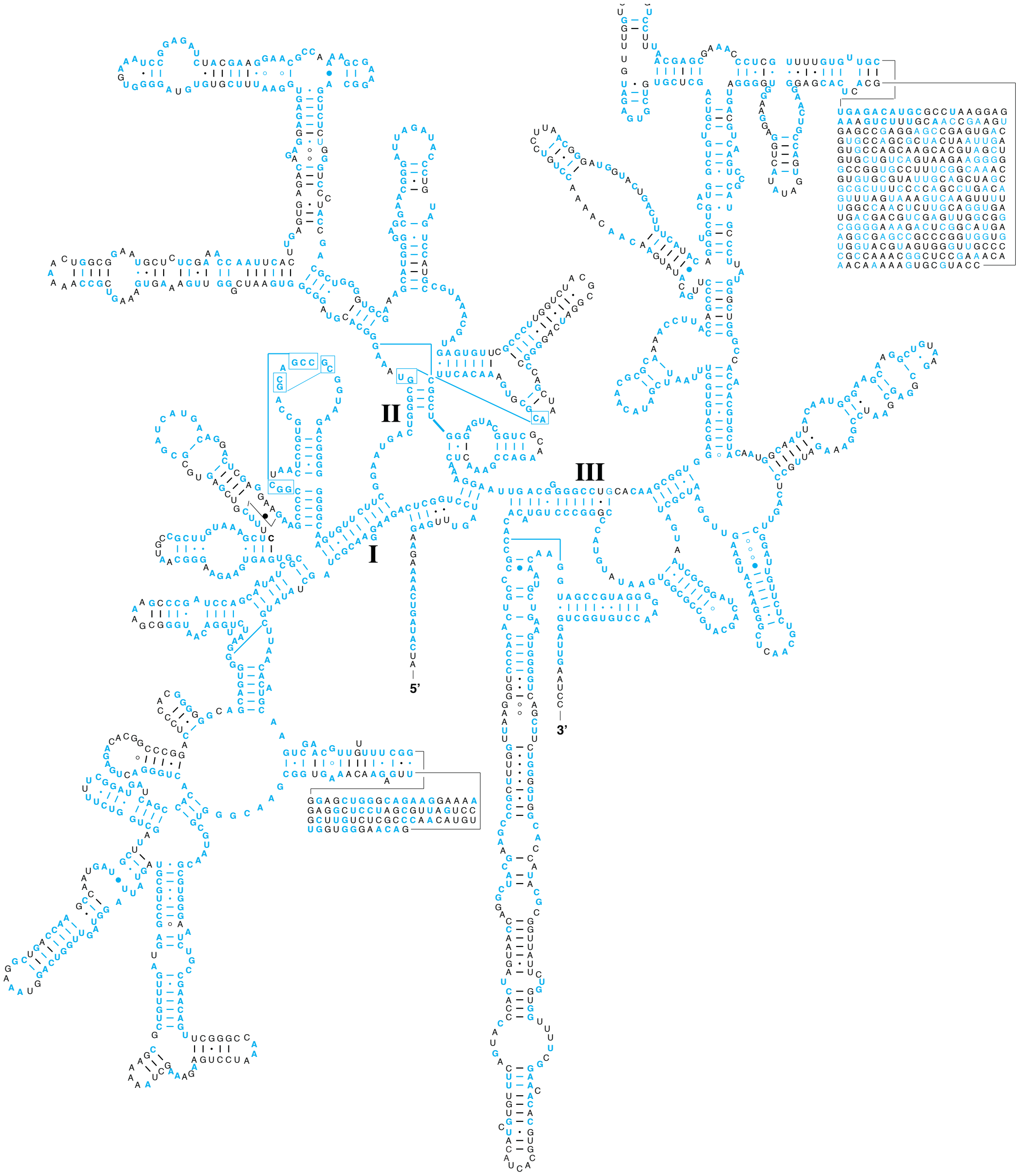

Fig. S3 Secondary structure model of *Oenothera berteriana* SSU mitochondrial rRNA. *Regions of sequence identity between* the SSU rRNAs of *O. berteriana* and *V. album* are indicated in blue. This secondary structure was downloaded from the Comparative RNA Website: http://www.rna.ccbb.utexas.edu [2].

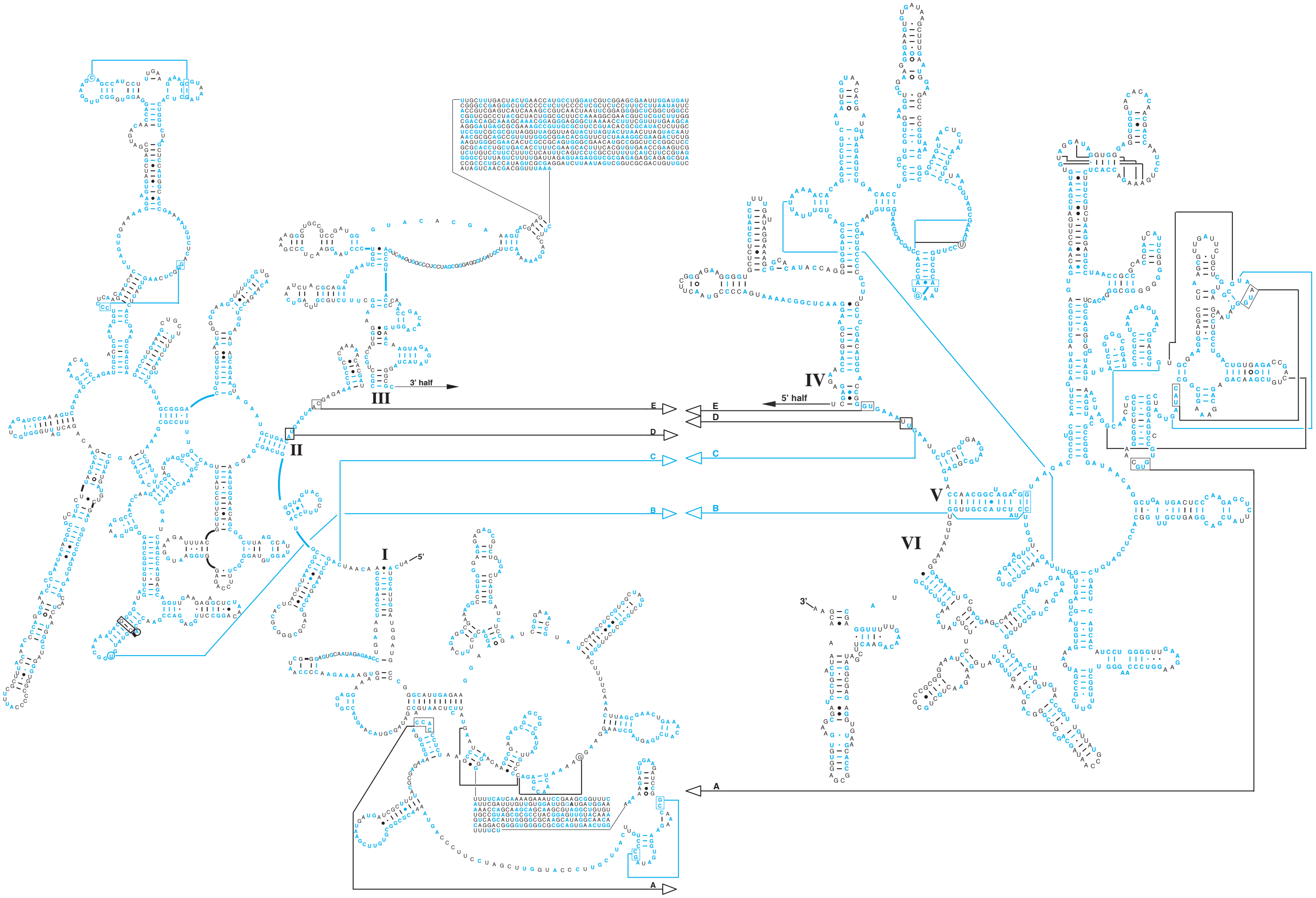

Fig. S4 Secondary structure model of *Zea mays* LSU mitochondrial rRNA. *Regions of sequence identity between* the LSU rRNAs of *Z. mays* and *V. album* are indicated in blue. This secondary structure was downloaded from the Comparative RNA Website: http://www.rna.ccbb.utexas.edu [2].

rrn5

```
                     1          11         21         31         41         51         61
                     |          |          |          |          |          |          |
Ancestor             AAACCGGGCA CTACGGTGAG ACGTGAAAAC ---ACCCGAT CCCATTCCGA CCTCG----A TATGTGGAAT
Amborella            .......... .......... .......... .......... .......... .......... ..........
Liriodendron         .......... .......... .......... .......... .......... .......... ..........
Monocots
Spirodela            .......... .......... .......... .......... .......... .......... ..........
Butomus              ...G...... ...G...TC. .......... .......... .......T.. A......... ..........
Phoenix              .......... .......... .......... .......... .......... .......... ..........
Oryza                .......... .......... .......... .......... .......... .....ATAT. ..........
Zea                  .......... .......... .....TTT.. .......... .......... .....ATAT. ..........
Rosids
Vitis                .......... .......... .......... .......... .......... .......... ..........
Gossypium            .......... .......A.. .......... .......... .......... .......... ..........
Carica               .......... .......... .......... .......... .......... .......... ..........
Arabidopsis          .......... .......... .......... .......... .......... .......... ..........
Brassica             .......... .......... .......... .......... .......... .......... ..........
Ricinus              .......... .......A.. .......... .......... .......... .......... ..........
Hevea                .......... .......A.. .......... .......... .......... .......... ..........
Vigna                -......... .......... .......... .......... .......... .......... ..........
Lotus                .......... .......... .......... .......... .......... .......... ..........
Malus                .......... .......... .......... .......... .......... .......... ..........
Citrullus            -......... .......... .......... .......... .......... .......... ..........
Cucurbita            .......... .......A.. .......... .......... .......... .......... ..........
Viscum scurruloideum G.C.TA.CT. .CC..A.CCC ..A..G..CG .CT..T.... T..TGGTG.T T.AA.....T CGAA..AG.G
Viscum album         G.C.TA.CT. .CC..A.CCC ..A..G..CG .CT..T.... T..TGGTG.T T.AA.....T CGAA..AG.G
Comandra             ---------- ----...... .........T GAA....... .......... .....ATGTG .G........
Amyema               -......... .......... .......... .......... .......... .......... ..........
Superasterids
Beta                 .......... .......... .......... .......... .......... .......... ..........
Silene noctiflora    ...GG..... A......A.. .....TC..G .....G.... A...C..G.. TT........ G....CA..C
Silene conica        G.CGT..AA. GG..T..... .T.GA.C..G .........G ....C..T.C .......... G....CA..C
Silene vulgaris      .......... ....A..... ...A..C..G .........G ....C..... T......... G....CA..C
Silene latifolia     .......... ....A..... ...A..C..G .........G ....C..... T......... G....CA..C
Helianthus           .......... .......... .......... .......... .......... .......... ..........
Daucus               .......... .......... .......... .......... .......... .......... ..........
Nicotiana            ...G...... .......A.. .......... .......... .......... .......... ..........
Asclepias            ---....... .......... .......... .......... .......... .......... ..........
Rhazya               .......... .......... .......... .......... .......... .......... ..........
Boea                 ---....... .......... .......... .......... .......... .......... ..........
Mimulus              .......... .......... .......... .......... .......... .......... ..........
Salvia               ---....... .......... .......... .......... .......... .......... ..........
Ajuga                ..G....... .C..A.C... .......G.. .......... ...GC..T.. .......... ..C...A...

                     71         81         91         101        111        121
                     |          |          |          |          |          |
Ancestor             CGTCTTGCGC CATATGTACT GAGATTGTTC GGGAGACATG GT-CAAAGCC CGGTG-----
Amborella            .......... .......... .......... .......... ....C..... ...G-.....
Liriodendron         .......... .......... .......... .......... .......... ..........
Monocots
Spirodela            .......... .......... ..A....... .......... .......... .....AA...
Butomus              .......... .......... ..A....... .......... ..A.C..... ..C..AATCA
Phoenix              .......... .......... ..A....... .......... .......... ..........
Oryza                .......... .......... ..A....... .......... .......... ...G.T....
Zea                  .......... .......... ..A....... .......... .......... ..CCCCTAAA
Rosids
Vitis                .......... .......... .......... .......... ....C..... .....AA...
Gossypium            .......... .......... .......... .......... ...A.....A ....-.....
Carica               .......... .......... .......... .......... ....C..... .....AA...
Arabidopsis          .......... .......... .......... .......... ....C..... .....A....
Brassica             .......... .......... .......... .......... ....T..... .....AA...
Ricinus              .......... .......... .......... .......... ....C..... ....AAA...
Hevea                .......... .......... .......... .......... ....C..... ....-.....
Vigna                .......... .......... ..A....... .......... .......... ....-.....
Lotus                .......... .......... ..A....... .......... ...A...... ....AAA...
Malus                .......... .......... ..A....... .......... ....C..... ...--.....
Citrullus            .......... .......... .......... .......... ....C..... .....AA...
Cucurbita            .......... .......... .......... .......... ....C..... .....AA...
Viscum scurruloideum .A.TGG.GC. AT........ ...-C..... .......... ..G.CGGAG. .AA.-.....
Viscum album         .A.TGG.GC. AT........ ...-C..... .......... ..G.CGGAG. .AA.-.....
Comandra             .......... .......... .......... .......... ....------ -----.....
Amyema               .......... .......... .......... .......... ....C..... ..........
Superasterids
Beta                 .......... .......... .......... .......... ....C..... .....AA...
Silene noctiflora    ..C...A... ........T. ..A....... .......... ...TCG.... .CC.TT....
Silene conica        .A...GA... .........C ......A... .......... ....TC.... ..CGTA....
Silene vulgaris      .......... .......... ......A... .......G.. ...G...... .----.....
Silene latifolia     .......... .......... ......A... .......G.. ...T.G.... ...CTA....
Helianthus           .......... .......... .......... .......... ....C..... ....-.....
Daucus               .......... .......... .......... .......... ....C..... .....AA...
Nicotiana            .......... .......... .......... .......... ....C..... .....AA...
Asclepias            .......... .......... .......... .......... ....C..... ....-.....
Rhazya               .......... .......... .......... .......... ....C..... .....AA...
Boea                 .......... .......... .......... .......... ...A...... ...A-.....
Mimulus              .......... .......... .......... .......... .......... ...--.....
Salvia               .......... .......... .......... .......... .......... ...--.....
Ajuga                .......... .......... .....AAC.. .......... .......... ...--.....
```

Fig. S5 Nucleotide alignment of the mitochondrial 5S rRNA gene. Taxa are shown in phylogenetic order. The ancestral sequence was reconstructed using baseml in the PAML package [3].

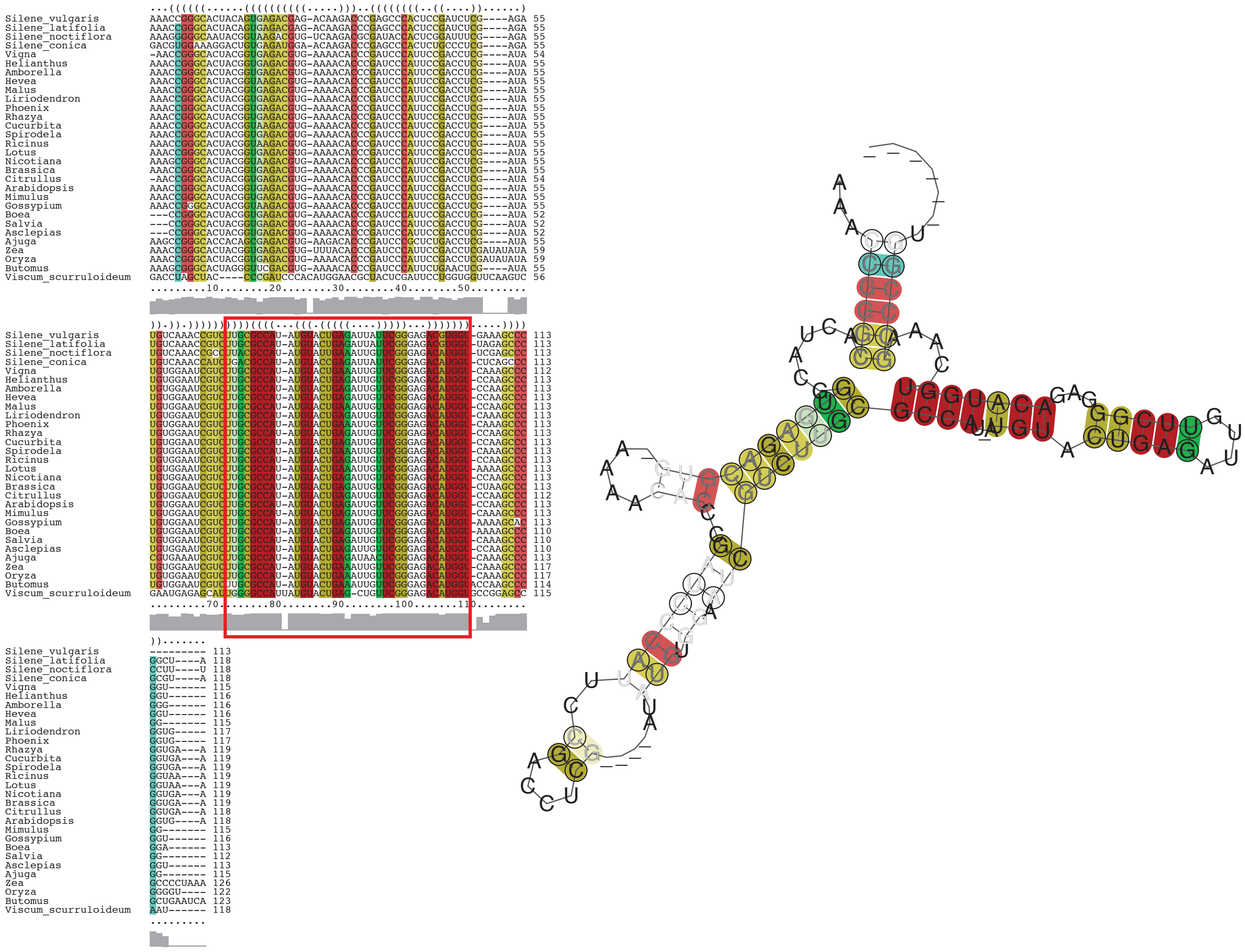

Fig. S6 Angiosperm 5S rRNA alignment within annotated consensus structure constructed using the LocARNA [4] tool for multiple alignment of RNA molecules. The red box indicates the original *V. scurruloideum* 5S rRNA annotation.

Tryptophan
(W)(CCA)
coordinates: 6553-6628
cove score: 52.48

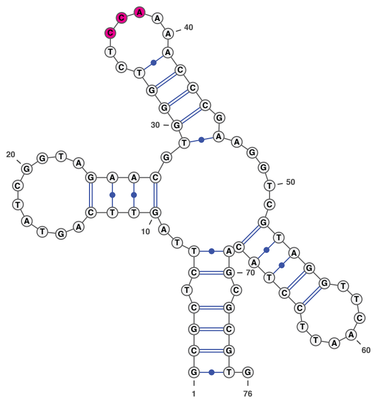

Methionine
(M)(CAT)
coordinates: 42653-42728
cove score: 40.43

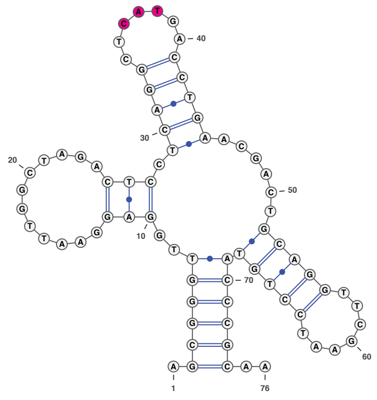

Lysine
(K)(TTT)
coordinates: complement(500766-500844)
cove score: 78.53

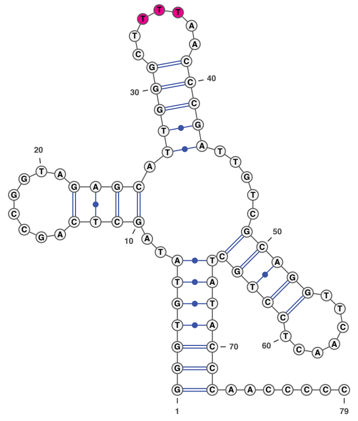

Tryptophan
(W)(CCA)
coordinates: 148987-149060
cove score: 70.10

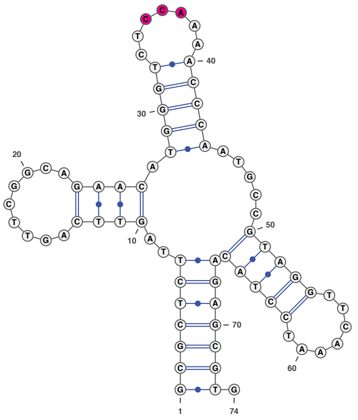

Methionine
(M)(CAT)
coordinates: complement(412180-412252)
cove score: 70.15

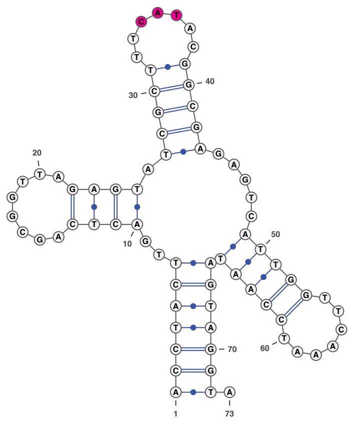

Glycine
(G)(GCC)
coordinates: complement(92533-92604)
cove score: 67.59

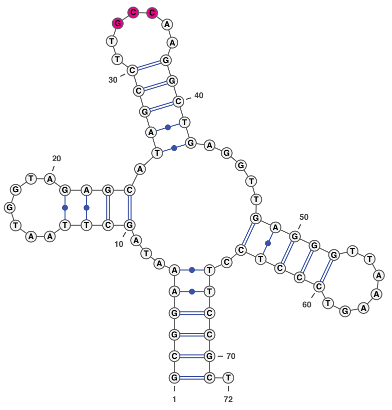

Fig. S7 *Viscum album* tRNAs. Anticodons are marked in pink. Secondary structures and anticodons were predicted using tRNAscan-SE [5]. Prediction scores are shown for each tRNA. Secondary structures were visualized using the VARNA java web start applet (http://varna.lri.fr/) [6].

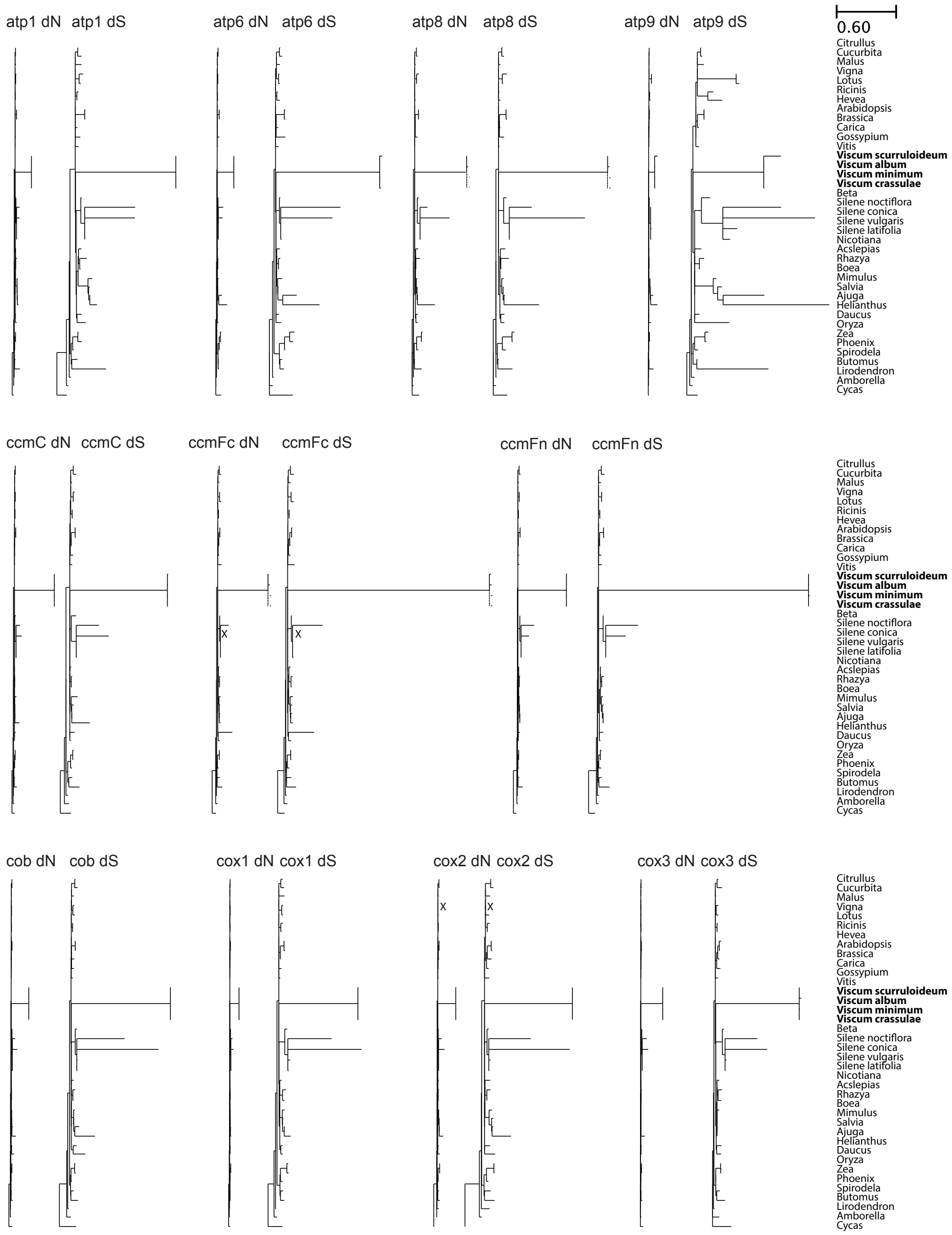

Fig. S8 Mitochondrial sequence divergence among 37 angiosperms and outgroup *Cycas*. Shown are topologically constrained phylograms of synonymous (d_S) and nonsynonymous (d_N) divergence for 17 of the 19 mitochondrial protein genes present in both *V. scurruloideum* and *V. album*. All trees are shown to the same scale. For "core" genes (*atp1-mttB*), an "X" indicates gene loss, and a dotted line indicates that a gene has not been annotated in *V. crassulae* and *V. minimum* by Petersen et al. (2015), but is present in both *V. scurruloideum* and *V. album,* and is probably present in the other two. For "variable" genes (*rpl16-rps12*), only species that contain the gene are shown. Phylograms for *atp4* and *sdh3* are not shown due to exceptionally high amino acid divergence.

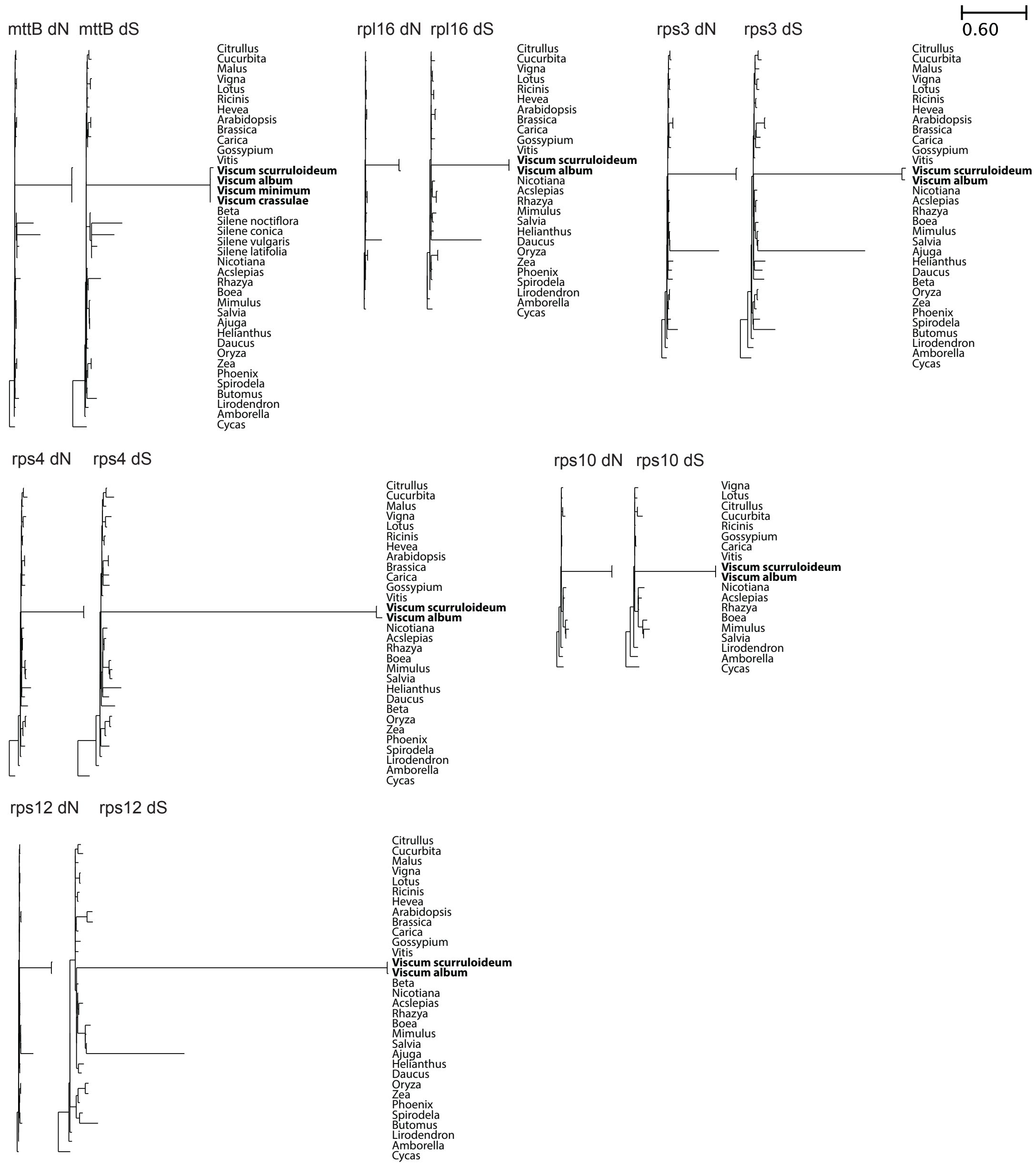

Fig. S8 Mitochondrial sequence divergence among 37 angiosperms and outgroup *Cycas*. Shown are topologically constrained phylograms of synonymous (d_S) and nonsynonymous (d_N) divergence for 17 of the 19 mitochondrial protein genes present in both *V. scurruloideum* and *V. album*. All trees are shown to the same scale. For “core” genes (*atp1-mttB*), an “X” indicates gene loss, and a dotted line indicates that a gene has not been annotated in *V. crassulae* and *V. minimum* by Petersen et al. (2015), but is present in both *V. scurruloideum* and *V. album,* and is probably present in the other two. For “variable” genes (*rpl16-rps12*), only species that contain the gene are shown. Phylograms for *atp4* and *sdh3* are not shown due to exceptionally high amino acid divergence.

References

1. Wertheim JO, Murrell B, Smith MD, Kosakovsky Pond SL, Scheffler K. RELAX: detecting relaxed selection in a phylogenetic framework. Mol Biol Evol. 2015;32:820-32.

2. Cannone JJ, Subramanian S, Schnare MN, Collett JR, D'Souza LM, Du Y, Feng B, Lin N, Madabusi LV, Müller KM *et al*. The comparative RNA web (CRW) site: an online database of comparative sequence and structure information for ribosomal, intron, and other RNAs. BMC Bioinformatics 2012;**3**:2.

3. Yang ZH. 2007. PAML 4: Phylogenetic analysis by maximum likelihood. Mol Biol Evol. 2007;24:1586-91.

4. Will S, Joshi T, Hofacker IL, Stadler PF, Backofen R. LocARNA-P: accurate boundary prediction and improved detection of structural RNAs. RNA 2012;18:900-14.

5. Lowe TM, Eddy SR. tRNAscan-SE: a program for improved detection of transfer RNA genes in genomic sequence. Nucleic Acids Res. 1997;25:955-64.

6. Darty K, Denise A, Ponty Y. VARNA: interactive drawing and editing of the RNA secondary structure. Bioinformatics 2009;25:1974-75.
